# Functional genomics of AP-2α and AP-2γ in cancers: in silico study

**DOI:** 10.1186/s12920-020-00823-9

**Published:** 2020-11-19

**Authors:** Damian Kołat, Żaneta Kałuzińska, Magdalena Orzechowska, Andrzej K. Bednarek, Elżbieta Płuciennik

**Affiliations:** Department of Molecular Carcinogenesis, Medical University of Lodz, 90-752 Lodz, Poland

**Keywords:** Cancer, Transcription factors, AP-2α, AP-2γ, Target genes, Bioinformatics

## Abstract

**Background:**

Among all causes of death, cancer is the most prevalent and is only outpaced by cardiovascular diseases. Molecular theory of carcinogenesis states that apoptosis and proliferation are regulated by groups of tumor suppressors or oncogenes. Transcription factors are example of proteins comprising representatives of both cancer-related groups. Exemplary family of transcription factors which exhibits dualism of function is Activating enhancer-binding Protein 2 (AP-2). Scientific reports concerning their function in carcinogenesis depend on particular family member and/or tumor type which proves the issue to be unsolved. Therefore, the present study examines role of the best-described AP-2 representatives, AP-2α and AP-2γ, through ontological analysis of their target genes and investigation what processes are differentially regulated in 21 cancers using samples deposited in Genomic Data Analysis Center (GDAC) Firehose.

**Methods:**

Expression data with clinical annotation was collected from TCGA-dedicated repository GDAC Firehose. Transcription factor targets were obtained from Gene Transcription Regulation Database (GTRD), TRANScription FACtor database (TRANSFAC) and Transcriptional Regulatory Relationships Unraveled by Sentence-based Text mining (TRRUST). Monocle3 R package was used for global samples profiling while Protein ANalysis THrough Evolutionary Relationships (PANTHER) tool was used to perform gene ontology analysis.

**Results:**

With RNA-seq data and Monocle3 or PANTHER tools we outlined differences in many processes and signaling pathways, separating tumor from normal tissues or tumors from each other. Unexpectedly, a number of alterations in basal-like breast cancer were identified that distinguished it from other subtypes, which could bring future clinical benefits.

**Conclusions:**

Our findings indicate that while the AP-2α/γ role remains ambiguous, their activity is based on processes that underlie the cancer hallmarks and their expression could have potential in diagnosis of selected tumors.

## Background

Following cardiovascular diseases, cancer is characterized by the second highest global mortality rate of any disease [1]. Many paradigms have been developed to explain the process of cancer development [2–4]—among them is the molecular theory of carcinogenesis associated with tumor suppressors and oncogenes [5], having its origin in the twentieth century [6]. It postulates that carcinogenesis is determined by alterations in cancer regulatory genes, of which two crucial groups are tumor suppressors and oncogenes, both responsible for apoptosis and proliferation regulation [7] being utmost importance in the model of cancer platform [8]. Nonetheless, occurrence of cancer-related genes demonstrating concurrently suppressive and oncogenic characteristics is definite for instance in transcription factors (TFs) [9]. Of these, probably the best known is Guardian of the Genome (p53 protein), whose correct activity requires that all of its subunits are not mutated and that no dominant negative effect occur [10]. In addition, the members of the Activating enhancer-binding Protein 2 (AP-2) family also demonstrate ambiguity; of these, AP-2α exhibits dualism of function depending on its related signaling pathway [11, 12]. Another AP-2 protein, AP-2γ, was thought to have oncogenic activity since its transcriptional activity is inhibited by WW Domain Containing Oxidoreductase (WWOX) tumor suppressor after its sequestration outside the nucleus [13]. However, the other reports indicating that AP-2γ induces expression of p21 protein suggest that it may also demonstrate anti-tumoral traits [14], presenting contrasting function which prompts consideration.

The entire AP-2 family fits within the basic Helix–Span–Helix (bHSH) superclass and comprises the five members (AP-2α, AP-2β, AP-2γ, AP-2δ, AP-2ε) encoded by *TFAP2A-E* genes [15]. The activation domain is located in the amino terminus, whereas the sequences responsible for dimerization and binding of DNA are on the side of carboxyl terminus [16]. All members recognize specific G/C-rich evolutionarily conserved motifs i.e. GCCN3/4GGC, GCCN3/4GGG or CCCCAGGC [17, 18] while the binding of transcription factors’ themselves is dictated by a proline-rich motif located in the activation domain (excluding AP-2δ, which lacks these critical residues) [15]. Cytogenetically, AP-2 family members are encoded on the sixth chromosome, except AP-2γ and AP-2ε which are on the twentieth and first chromosomes, respectively [16]. When functioning correctly, the AP-2 transcription factors regulate appropriate gene expression during early developmental processes such as face, eye or limb development [19]. It was suggested that in the case of mutation, the loss of TF activity of AP-2 members can lead to the impairment of proliferation, differentiation and apoptosis processes [16], suggesting AP-2 activity may play role in development. Indeed, both AP-2α and AP-2γ have prognostic value for some tumor types. AP-2α overexpression is recognized as a prognostic indicator of shorter patient survival in papillary thyroid carcinoma and epithelial ovarian cancer but longer survival in gastric adenocarcinoma [20–22]. In terms of AP-2γ, its overexpression was also associated with poorer overall survival in breast cancer patients [23] with the other literature data to support this statement and enrich it with correlation presenting worse anti-hormone therapy response [24]. The other case of chemoresistance (to 5-fluorouracil) was shown in colorectal cancer upregulating AP-2γ [25] while endometrial cancer example demonstrated that knockdown of this AP-2 member sensitizes cells to megestrol acetate via Estrogen receptor alpha (ERα) expression upregulation [26].

Literature data indicates that the activity of AP-2 in carcinogenesis is very much dependent on specific family members. The most recent scientific reports concern the first three transcription factors, while the least is known about AP-2δ or AP-2ε. The best described factors, AP-2α and AP-2γ, play distinct roles depending on tumor type, as noted in a recent review [15]; however; this summary has since been enriched with additional tumor models not available at that time. These findings confirm that AP-2α activity is influenced by tumor tissue type: while it was found to demonstrate oncogenic activity in cervical, gallbladder and ovarian cancer [27–29], it has been also seen to act as a suppressor in many other tumors [11, 30–35]. AP-2γ has been conclusively demonstrated to play an oncogenic role in breast cancer [36] (which coincides with previous evaluation) and furthermore expanded with its unfavorable characteristics in colorectal cancer [25]. The last representative of the AP-2 family abundantly described in the literature is beta member. Latest data regarding contribution in cancer indicates that AP-2β overexpression has been found to promote tumor growth in both breast and thyroid cancer and predicted poor prognosis or tumor progression, respectively [37, 38]. Finally, although little is known of AP-2δ and AP-2ε, the former is thought to be associated with progression and genomic instability of prostate cancer while the latter acts as a tumor suppressor in neuroblastoma [39, 40].

The precise functionality of AP-2 factors in cancer clearly remains unknown. Therefore, the aim of the present study was to clarify the role of transcription factors AP-2α and AP-2γ in carcinogenesis using samples (RNA-seq) acquired from 21 cancers of The Cancer Genome Atlas (TCGA) and Monocle3 or Protein Analysis Through Evolutionary Relationships (PANTHER) bioinformatics tools. The study identifies the processes which are differentially regulated between the studied cancer types, compare the findings with those of normal tissue and identifies whether any differences exist in the expression of target genes for these factors in specific types of tumors.

## Methods

### Acquisition of tumor patients’ data

RNA-seq expression data with corresponding clinical annotation was collected from 21 tumors (Table 1) of TCGA-dedicated GDAC Firehose Repository (level 3 RNA-seqV2, RSEM normalized, data status of 28th Jan 2016 available at https://gdac.broadinstitute.org/). Patients that lacked expression or clinical data were discarded from the study. Available normal, paired solid tissues were additionally retrieved through R-dedicated package TCGA-Assembler [41].

**Table 1:** Cohorts selected from GDAC Firehose Repository

Cohort	Description
BLCA	Bladder urothelial carcinoma
BRCA	Breast invasive carcinoma
CESC	Cervical and endocervical cancers
COAD	Colon adenocarcinoma
ESCA	Esophageal carcinoma
GBM	Glioblastoma multiforme
HNSC	Head and neck squamous cell carcinoma
KIRC	Kidney renal clear cell carcinoma
KIRP	Kidney renal papillary cell carcinoma
LGG	Brain lower grade glioma
LIHC	Liver hepatocellular carcinoma
LUAD	Lung adenocarcinoma
LUSC	Lung squamous cell carcinoma
OV	Ovarian serous cystadenocarcinoma
PAAD	Pancreatic adenocarcinoma
PRAD	Prostate adenocarcinoma
READ	Rectum adenocarcinoma
SKCM	Skin cutaneous melanoma
STAD	Stomach adenocarcinoma
THCA	Thyroid carcinoma
UCEC	Uterine corpus endometrial carcinoma

### AP-2α and AP-2γ target genes identification

Targets for both transcription factors were identified through several databases: GTRD (version 19.10 [42, 43]), TRANSFAC (version 2019.2) and TRRUST (version v2). Excluding duplicates, there were 4810 and 5175 targets for AP-2α and AP-2γ, respectively.

### Global profiling of the tumors and ontological annotation

**Fig. 1:**
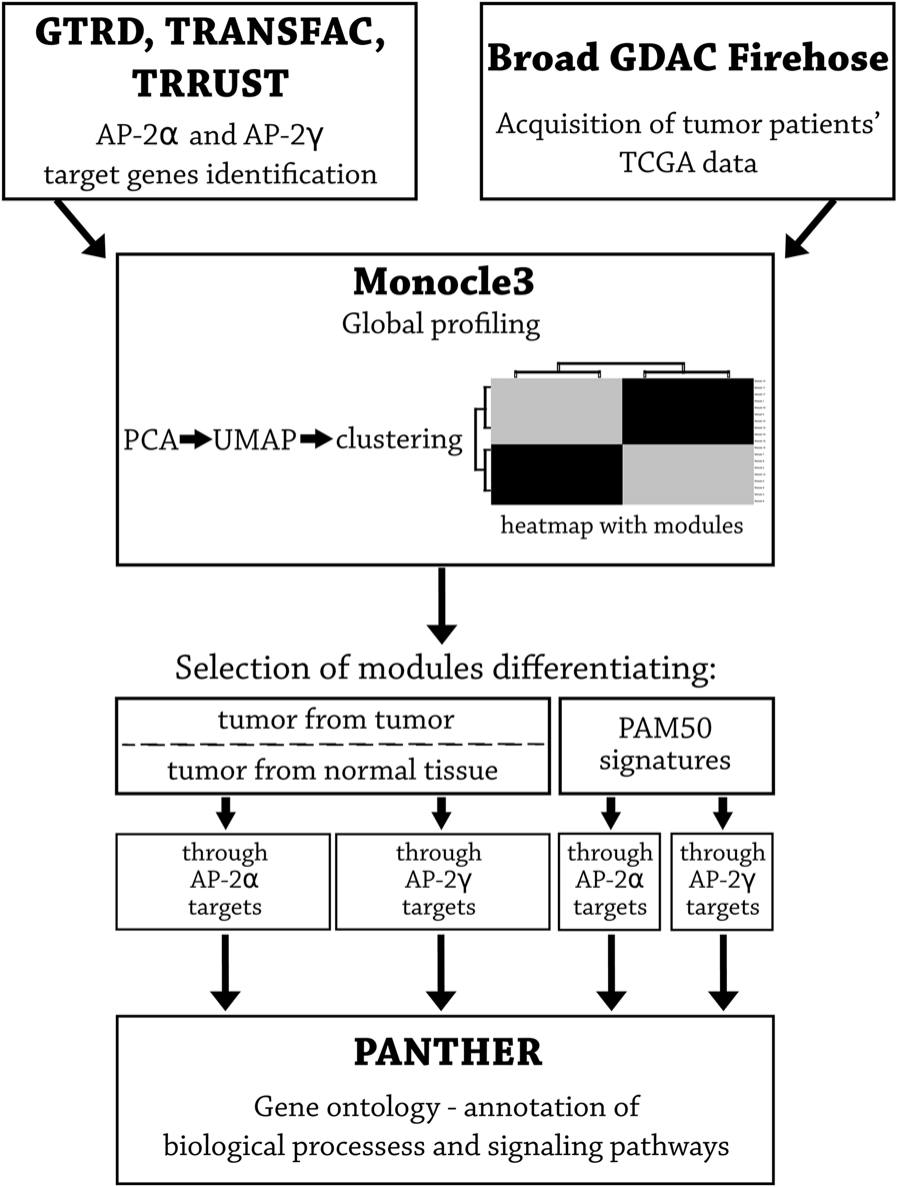
Visualization of the methodology

Phenotype heterogeneity between selected tumors accompanied by normal tissues was studied and visualized by applying the UMAP method preceded by principle component analysis (PCA) regarding the expression of the targets of both AP-2α and AP-2γ using the Monocle3 R package (https://cole-trapnell-lab.github.io/monocle3/) [44]. The analyses of AP-2α and AP-2γ targets were performed as two separate entities accordingly. The PCA pre-processing step (preprocess_cds()) was done with the dimensionality of the reduced space of 100 (num_dim). Reduction of dimensions (reduce_dimension()), and clustering of the individuals (cluster_cells()) within spaces were applied with the UMAP algorithm for dimensionality reduction method upon which to base clustering (reduction_method). The clusters of individuals were compared with graph_test() function based on Moran's I spatial autocorrelation analysis with knn neighbor graph and q-value threshold of 0.05. Furthermore, the genes varying across the clusters selected at the previous step were grouped into modules through Louvain community analysis (find_gene_modules()) with parameters set to default. The modules were clustered in two ways: 1) clustering involving all of the individuals enabling to compare between tumors and tumor vs non-tumor samples of specific cancer type and 2) clustering of the modules restricted to the BRCA foci enabling to differentiate between PAM50 subtypes, separately for AP-2α and AP-2γ targets. Finally, the results were visualized with pheatmap() and clustered with the Ward D2 method. The whole pipeline was performed according to the Monocle3 tutorial (https://cole-trapnell-lab.github.io/monocle3/). To annotate the findings in the context of the biological processes in which they are involved, overrepresentation test available at PANTHER Classification System (http://www.pantherdb.org/) with Fischer's Exact Test and Bonferroni correction for multiple testing were performed. The methodology is graphically summarized in Fig. 1.

## Results

### Identification of global differences using toolkit for spatial analysis

**Fig. 2:**
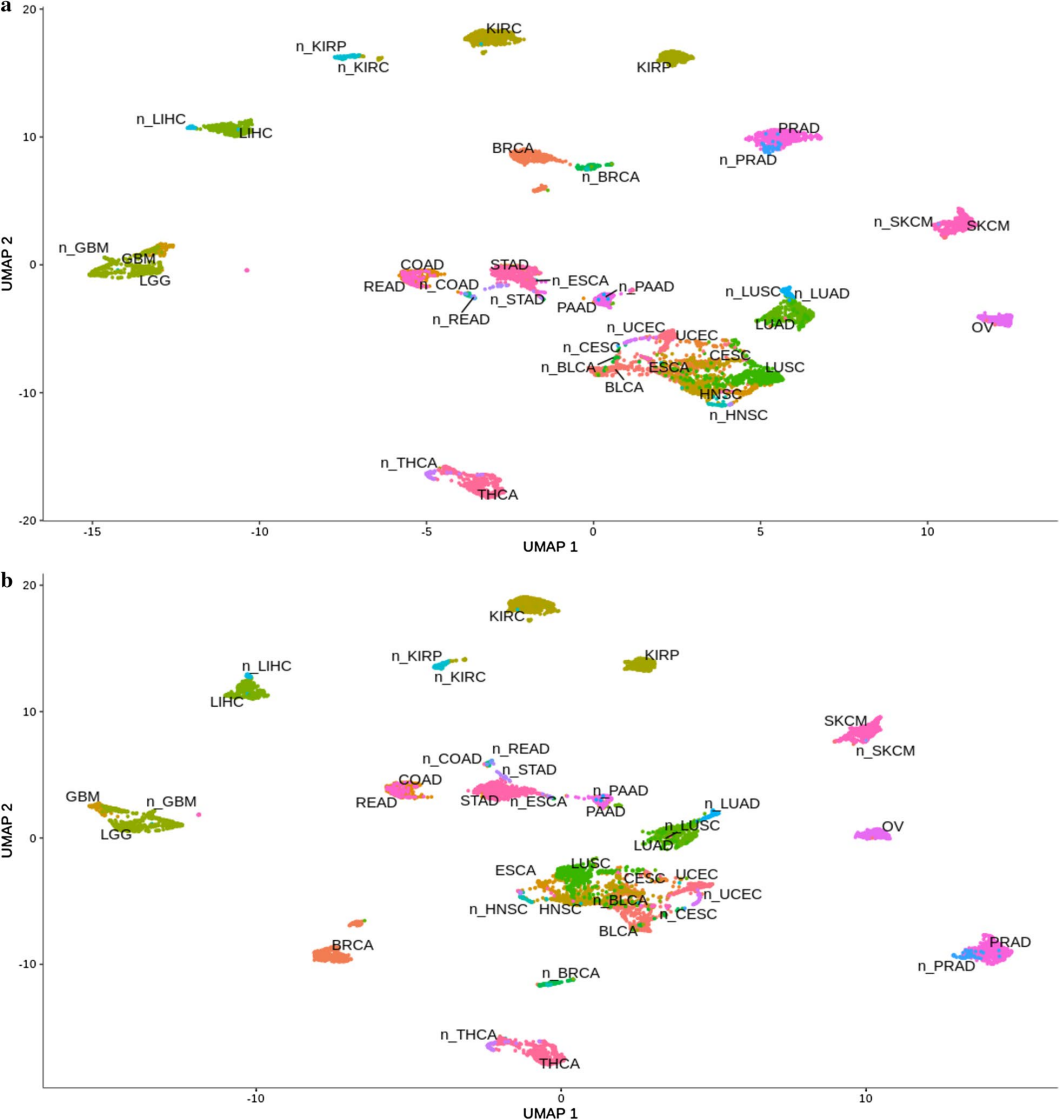
Spatial analysis showing differences between tumors and corresponding normal tissues. **a** AP-2α target gene list. **b** AP-2γ target gene list

Considered cohorts were clustered according to designed variable discriminating tumors and their corresponding normal tissues (marked with an additional prefix "n_"). Their distribution across Uniform Manifold Approximation and Projection (UMAP) dimensions was presented according to the genetic targets for a given transcription factor (AP-2α or AP-2γ). The analysis based on AP-2α identified a group of cancers distinct from other separated clusters but to a lesser extent from the corresponding normal tissue: LIHC, KIRP, KIRC, PRAD, SKCM, OV, THCA, BRCA, STAD, LUAD and PAAD. In some cases, one cancer type coincided in the plot with another (forming a “cluster”) e.g. GBM and LGG, COAD and READ. Surprisingly, the remaining tumor reservoir (BLCA, CESC, UCEC, ESCA, HNSC, LUSC) formed a heterogeneous mixed cluster together with their normal tissues, albeit with deviations for ESCA and LUSC. The same tendencies as of AP-2α were observed in terms of AP-2γ targets, although with minor differences—these are collectively presented in Fig. 2.

**Fig. 3:**
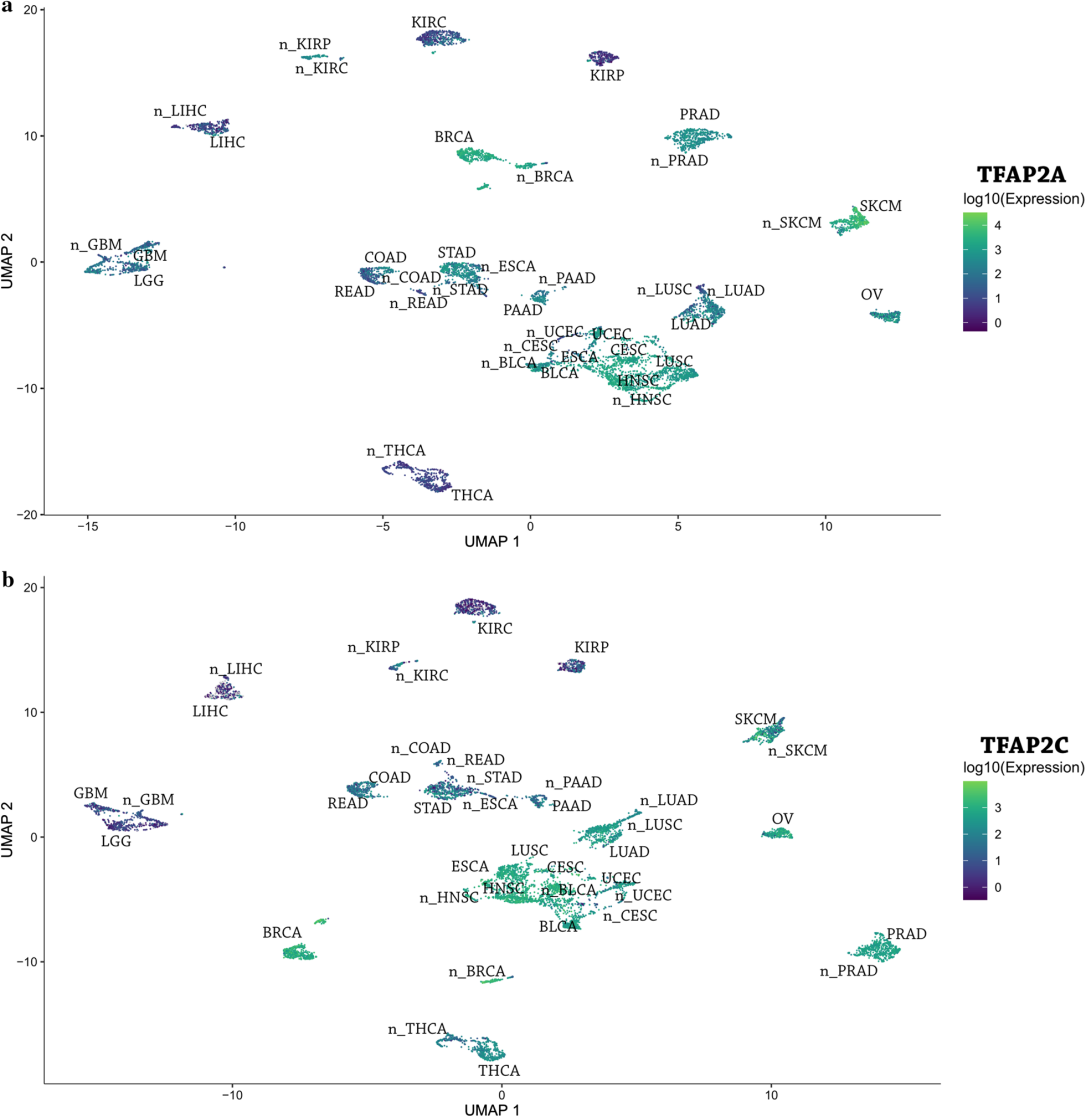
Differences in expression of TF-encoding gene among tumor types and corresponding normal tissues. **a** TFAP2A. **b** TFAP2C

In the next stage, distribution in the UMAP1 and UMAP2 dimensions was further analyzed with regard to differences in expression of the transcription factor itself. Global similarities were observed in the case of both AP-2α or AP-2γ expression; however, some exceptions were observed e.g. AP-2α expression was higher in SKCM while AP-2γ was higher in THCA (Fig. 3a, b, respectively). Evident differences in expression were observed between kidney tumors (KIRC, KIRP) and normal kidney tissue; this is not surprising in the case of AP-2α which has been suggested as a biomarker in renal carcinoma [45, 46] however our findings suggest that AP-2γ may have the same properties.

**Fig. 4:**
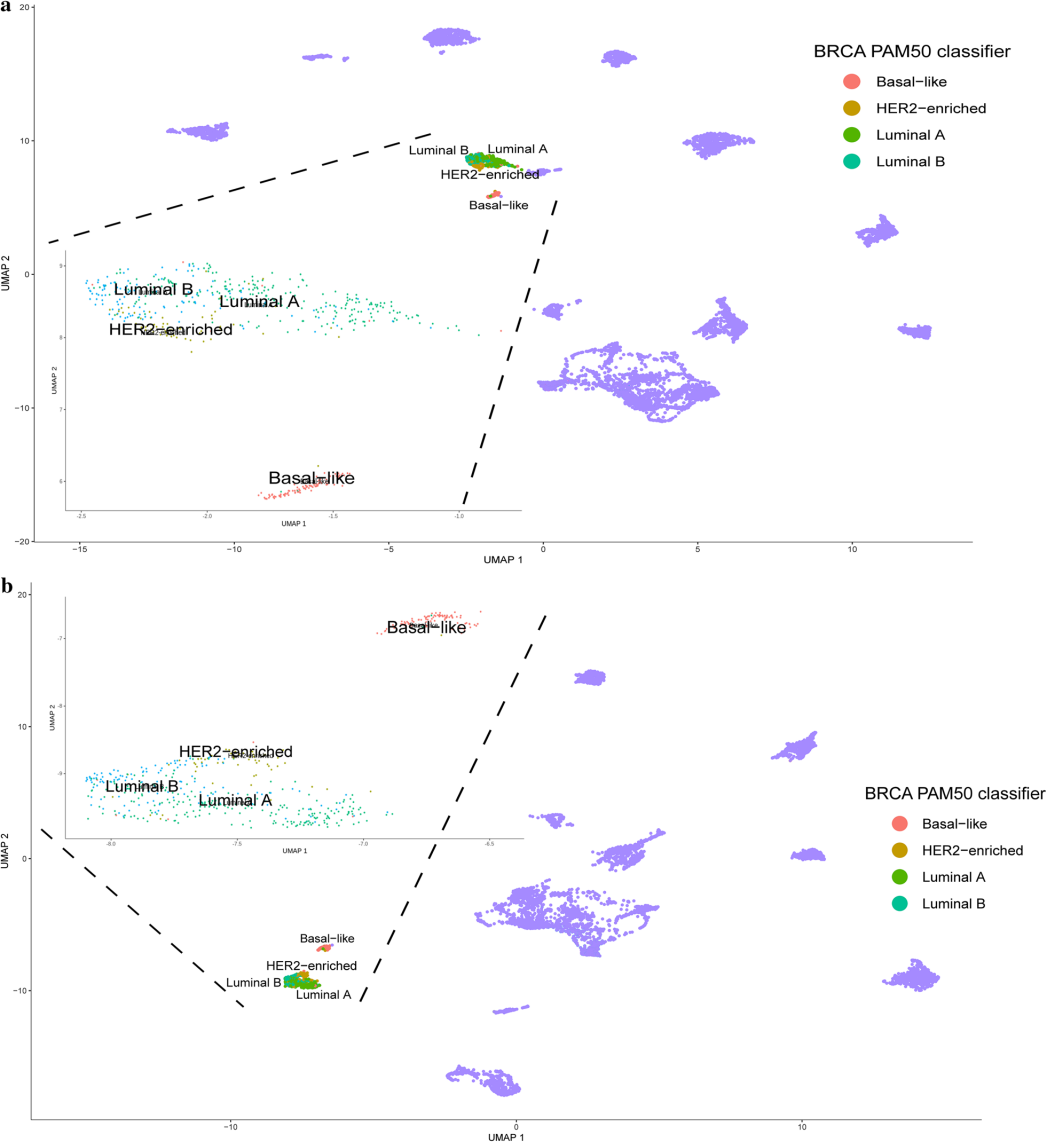
Spatial analysis showing differences between BRCA subtypes using target genes of a particular transcription factor. **a** AP-2α. **b** AP-2γ

Furthermore, due to noticeable separation of breast cancer samples visible for both target gene lists (Fig. 2), possession of extraordinary tumor profiling signature (Prosigna Breast Cancer Prognostic Gene Signature Assay—PAM50) prompted us to perform additional analyses to assess the subtypes’ separation by TFs target genes. For both AP-2α or AP-2γ, differences were observed in the PAM50 classifiers of breast cancer indicating that the basal-like subtype was different from the others (Fig. 4a, b, respectively).

**Fig. 5:**
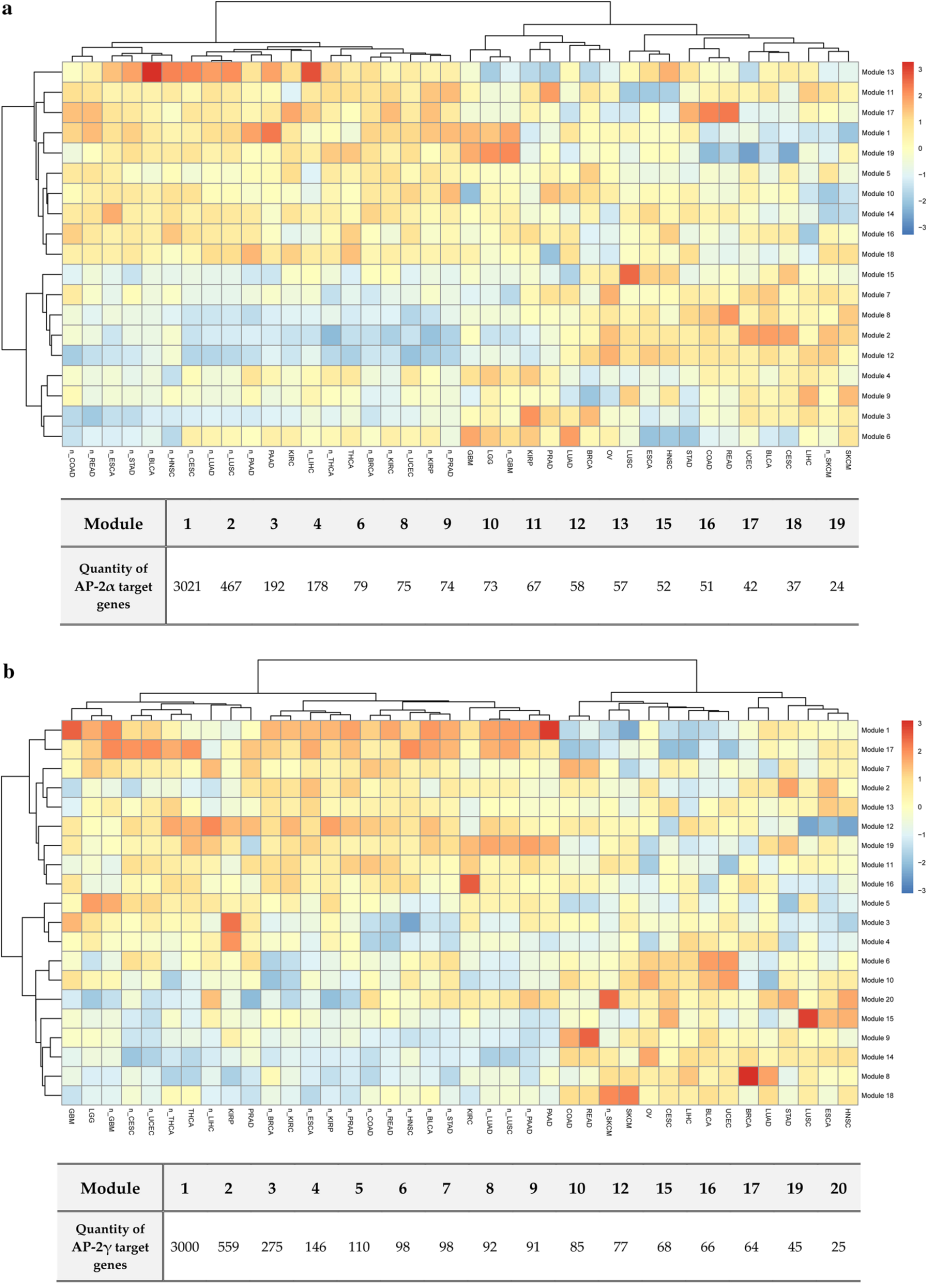
Heatmap presenting modules of transcription factor target genes differentiating tumors and corresponding normal tissues. **a** AP-2α targets. **b** AP-2γ targets. The prefix "n_" was added to indicate non-cancer tissue corresponding to the appropriate tumor type

**Fig. 6:**
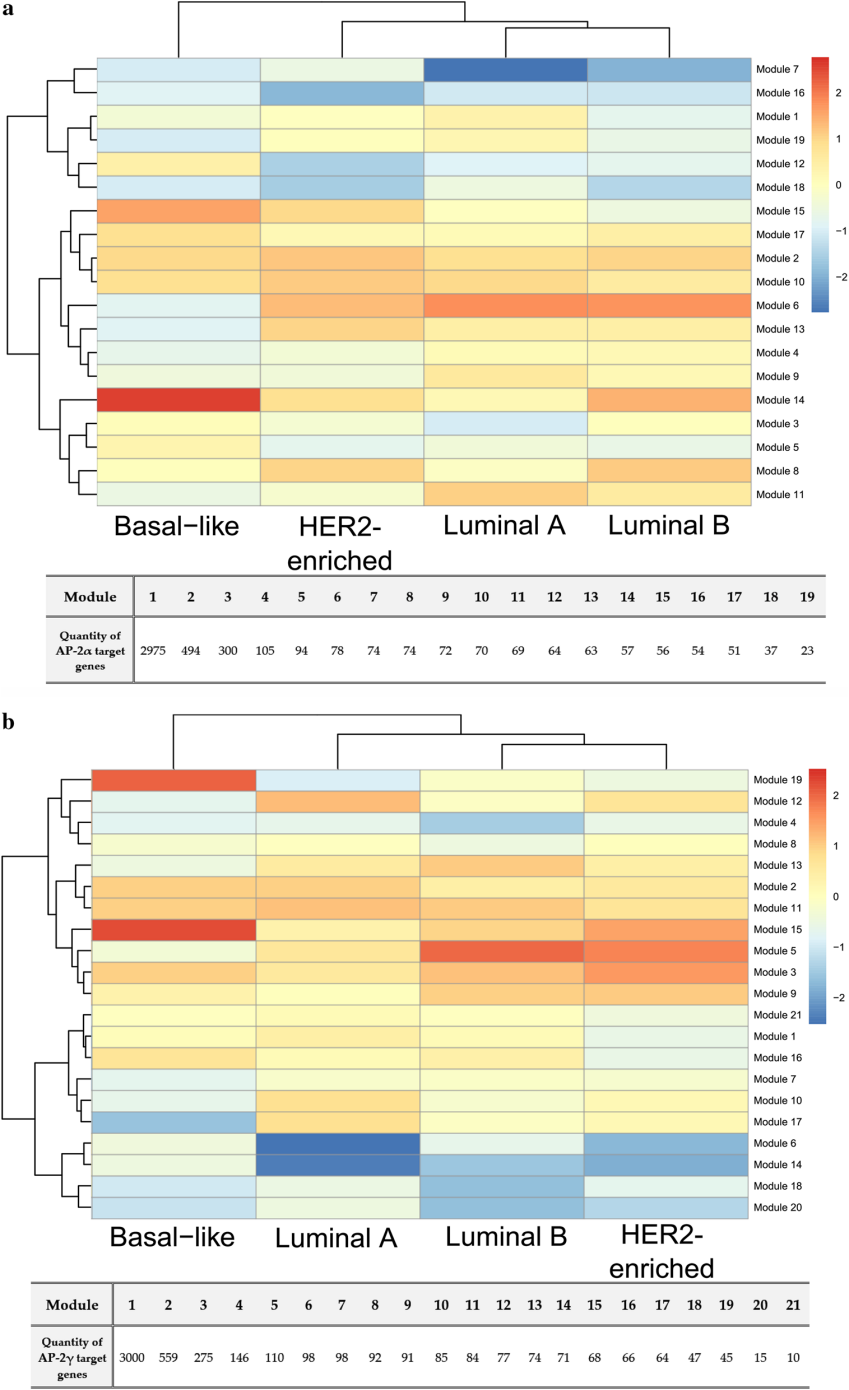
Heatmap presenting modules of transcription factor target genes differentiating PAM50 BRCA subtypes. **a** AP-2α targets. **b** AP-2γ targets

Lastly, heatmaps were generated to arrange the transcription factor target genes into modules with common expression profile, indicating how these modules differ between clusters; this approach allowed more specific differences between individual cancers, the tumor and the corresponding normal tissue or intrinsic subtypes to be identified (Figs. 5, 6). The content of modules from particular analysis type has been summarized in the Additional file 1. At first glance, signaling by the AP-2 targets in tumor tissue is congruent to signaling in the corresponding normal tissue for PAAD, LIHC, THCA and SKCM (in the case of AP-2α) or for PAAD, THCA and SKCM (in the case of AP-2γ). However, the analysis of signaling through TF targets in the remaining clusters indicated a large number of modules that could be used to distinguish the tumor from normal tissue (the latter taken as a reference), or the basal-like BRCA subtype from the luminal A/B and HER2-enriched subtypes. The proposed modules taken for further comparisons between tumor and normal tissue or between different tumor tissues are summarized in Table 2 (AP-2α) and Table 3 (AP-2γ). In the case of differences between BRCA subtypes, the modules 6, 12 and 13 were taken for subsequent consideration for the AP-2α target gene list while 5, 13, 19 modules for AP-2γ.

**Table 2:** Proposed modules distinguishing the tumor from corresponding normal tissue by means of AP-2α targets

Cancer tissue versus normal tissue	Module
KIRP versus n_KIRP	1
CESC versus n_CESC/UCEC versus n_UCEC	2
BRCA versus n_BRCA	3
LUAD versus n_LUAD	6
READ versus n_READ	8
GBM versus n_GBM	10
HNSC versus n_HNSC/KIRC versus n_KIRC	11
COAD versus n_COAD/ESCA versus n_ESCA/STAD versus n_STAD	12
BLCA versus n_BLCA	13
LUSC versus n_LUSC	15
PRAD versus n_PRAD	18

**Table 3:** Proposed modules distinguishing the tumor from corresponding normal tissue by means of AP-2γ targets

Cancer tissue versus normal tissue	Module
BLCA versus n_BLCA	1
STAD versus n_STAD	2, 5
KIRP versus n_KIRP	3
BRCA versus n_BRCA	8
KIRC versus n_KIRC	12, 20
ESCA versus n_ESCA/LUAD versus n_LUAD	12
LUSC versus n_LUSC	12, 15
HNSC versus n_HNSC	12, 17
GBM versus n_GBM/CESC versus n_CESC/UCEC versus n_UCEC	17
LIHC versus n_LIHC/PRAD versus n_PRAD	19
COAD versus n_COAD/READ versus n_READ	1, 3, 9

Another approach was to distinguish different cancer types with similar sites of origin. The following comparisons were used for gene modules generated from AP-2α and AP-2γ targets: COAD versus READ, LGG versus GBM, LUSC versus LUAD and KIRC versus KIRP. For AP-2α, COAD and READ gave similar results; however, LGG versus GBM were differentiated by modules 10 and 13, LUSC versus LUAD for modules 6, 9, 11, 15 and KIRC versus KIRP for modules 1, 3, 4, 6, 13, 16, 19. Regarding AP-2γ, COAD versus READ were differentiated by module 9, LGG versus GBM with module 5, LUSC versus LUAD with modules 7, 10, 12, 15 and KIRC versus KIRP with modules 3, 4, 12, 16.

The differences between tumors were then identified from a heterogeneous mixed cluster. Using modules 2, 11, 19 with target genes for AP-2α, the entire selection was divided into two different “communities”—LUSC, ESCA, HNSC and UCEC, BLCA, CESC. The same tendency could be explained using module 17 of AP-2γ targets with additional dissimilarities in UCEC versus LUSC or ESCA versus BLCA which can be explained by the inverse of module 15 or 6, respectively.

### Gene ontology of modules distinguishing specific comparisons

Analyzing a multitude of comparisons in the form of modules diversifying groups (from the previous section) led to the extraction of genes for their subsequent ontological analysis. The greatest attention was paid to modules showing the opposite tendency in the context of expression of genes contained therein. As previously, the following distinctions were made: between the cancer and the corresponding normal tissue, between similar tumors or between PAM50 classifiers for breast cancer.

#### Comparisons by means of target genes for AP-2α transcription factor

**Fig. 7:**
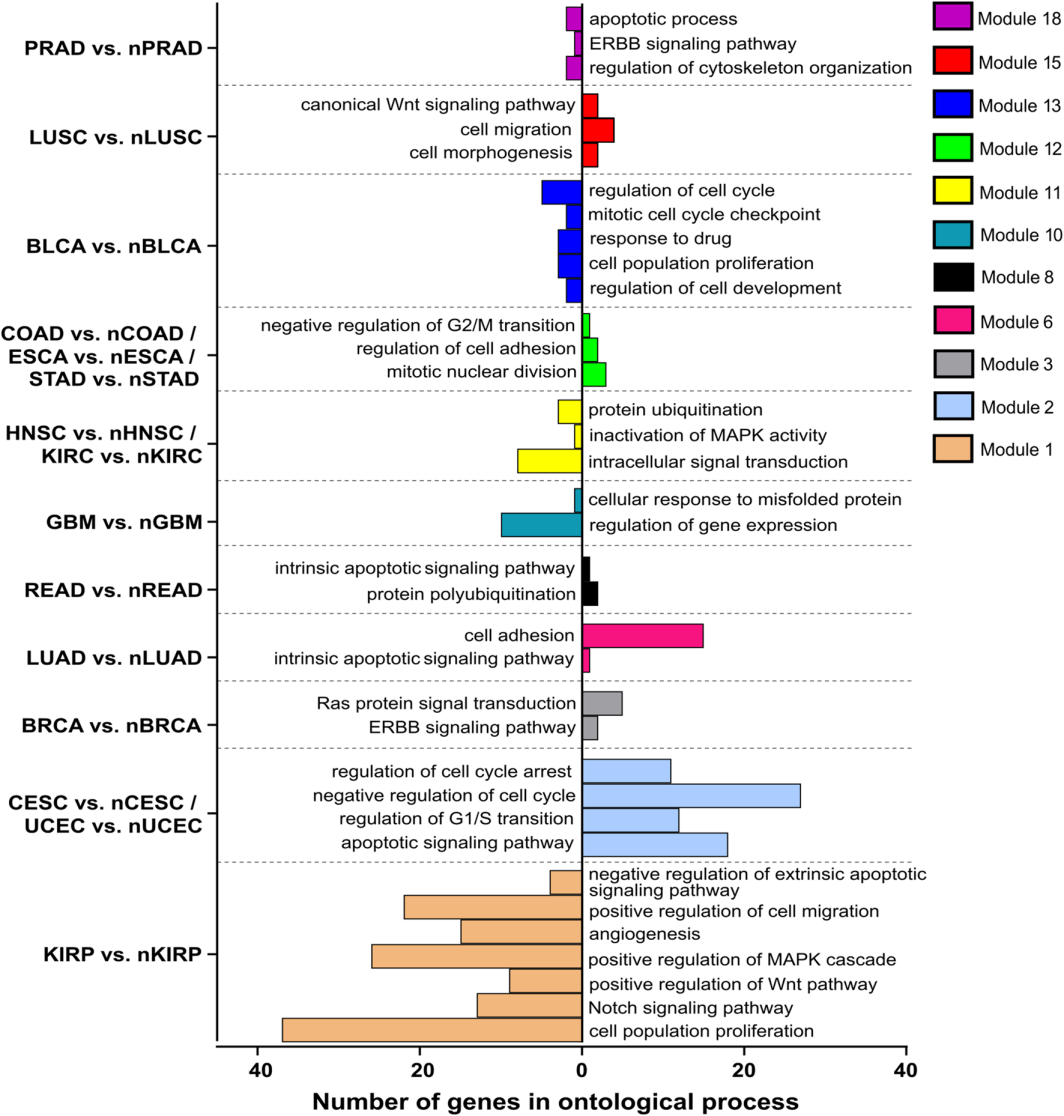
Gene ontology of selected modules differentiating tumor and corresponding tissue by means of AP-2α targets

**Fig. 8:**
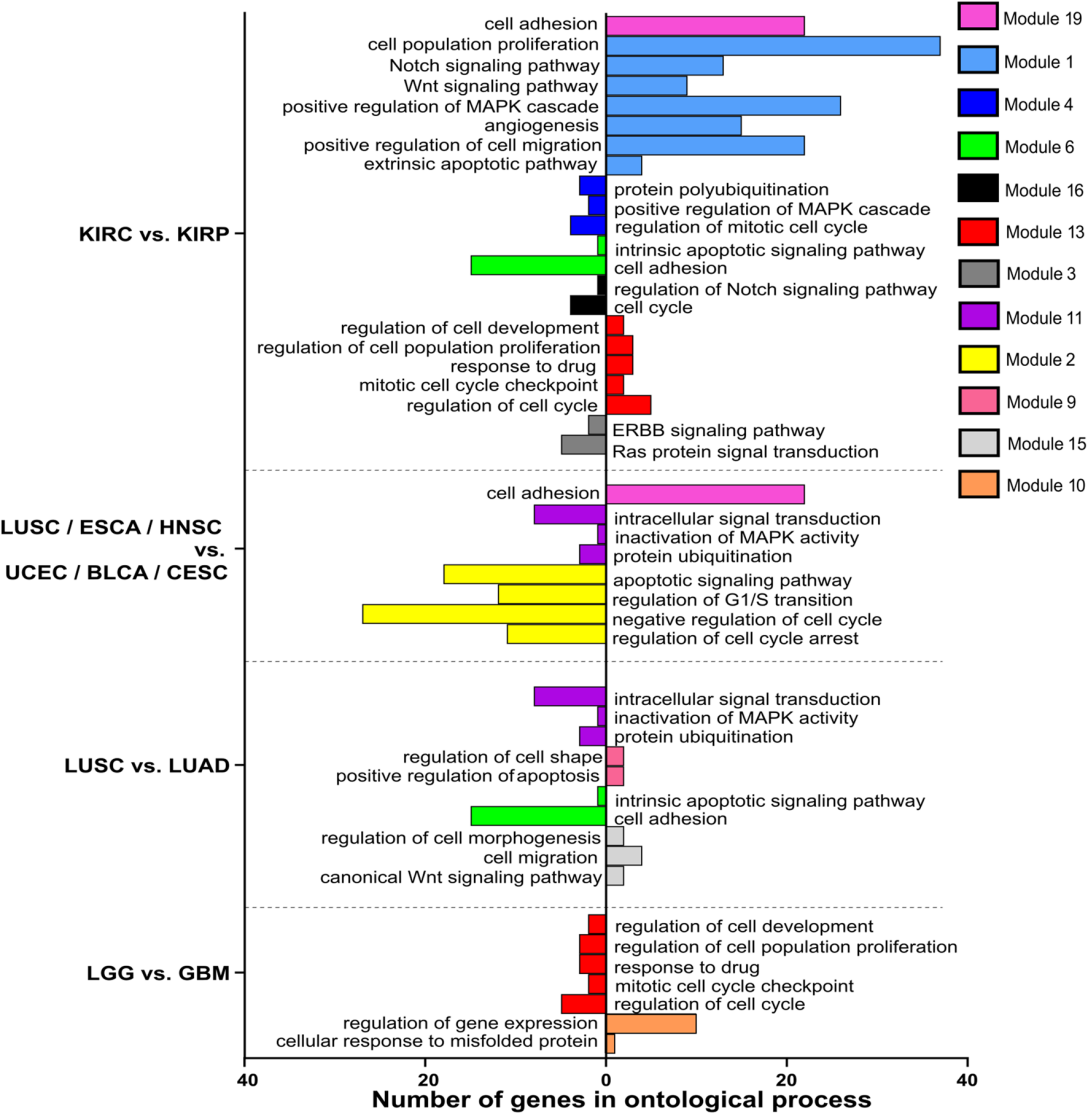
Gene ontology of selected modules differentiating tumors by means of AP-2α targets

Differences between tumors and normal tissues indicated changes in processes (e.g. apoptosis, proliferation, cell migration, cell cycle, cytoskeleton organization, cell adhesion or angiogenesis) and signal transduction pathways (e.g. ERBB, Wnt, MAPK and Notch) which are visualized in Fig. 7. In regards to alterations between different types of tumors, they were approximate to above mentioned however additional processes such as regulation of cell shape were also noticeable (Fig. 8). Further data such as specific genes that described a particular module during ontological analysis, as well as statistical significance, are reviewed in Additional file 2.

#### Comparisons by means of target genes for AP-2γ transcription factor

**Fig. 9:**
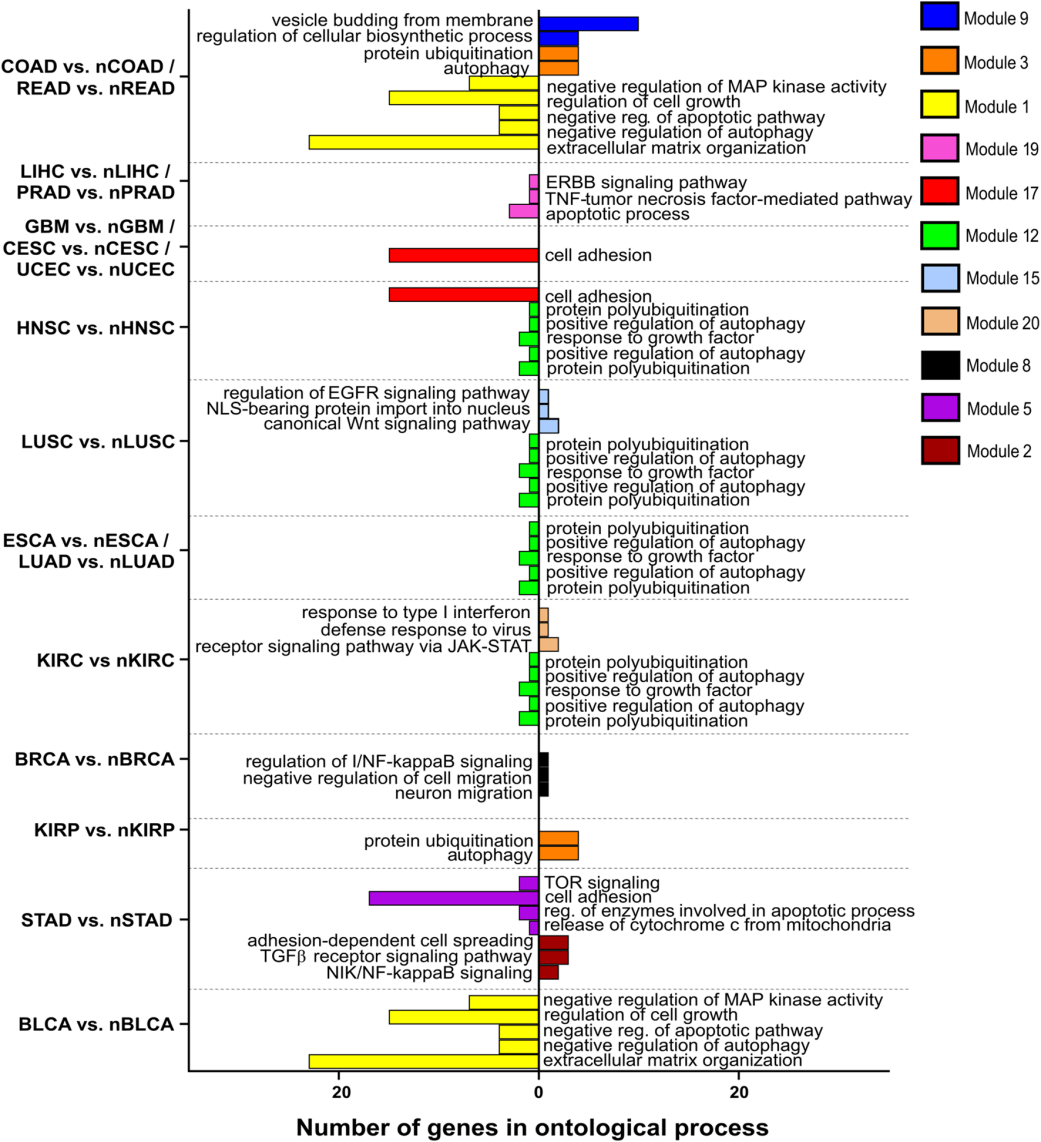
Gene ontology of selected modules differentiating tumor and corresponding tissue by means of AP-2γ targets

**Fig. 10:**
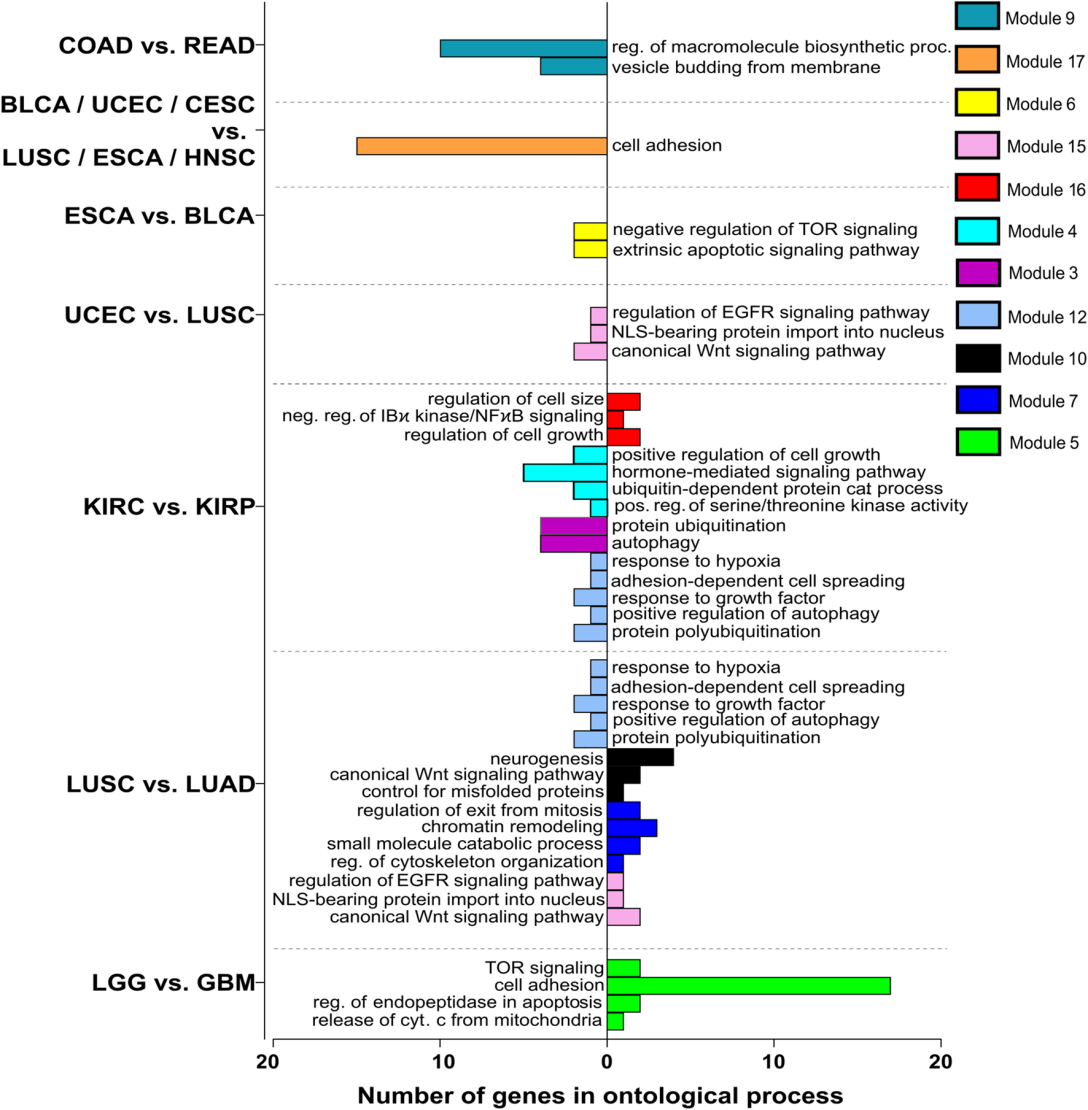
Gene ontology of selected modules differentiating tumors by means of AP-2γ targets

In the case of second AP-2 family representative, tumor versus normal tissue changes also affected processes (e.g. autophagy, adhesion, vesicle budding, extracellular matrix organization, apoptosis, cell growth and spreading) and signaling pathways (e.g. TNF, EGFR, Wnt, JAK-STAT, mTOR, NFkB and TGFβ) which is summarized in Fig. 9. Regarding variation between tumors, no additional processes other than those mentioned above were identified, even though different modules were used (i.e. module 6) (Fig. 10). The specific genes that characterizing individual modules through ontological analysis, together with their statistical significance, are recapitulated in Additional file 3.

#### Comparisons of breast cancer intrinsic PAM50 subtypes

**Fig. 11:**
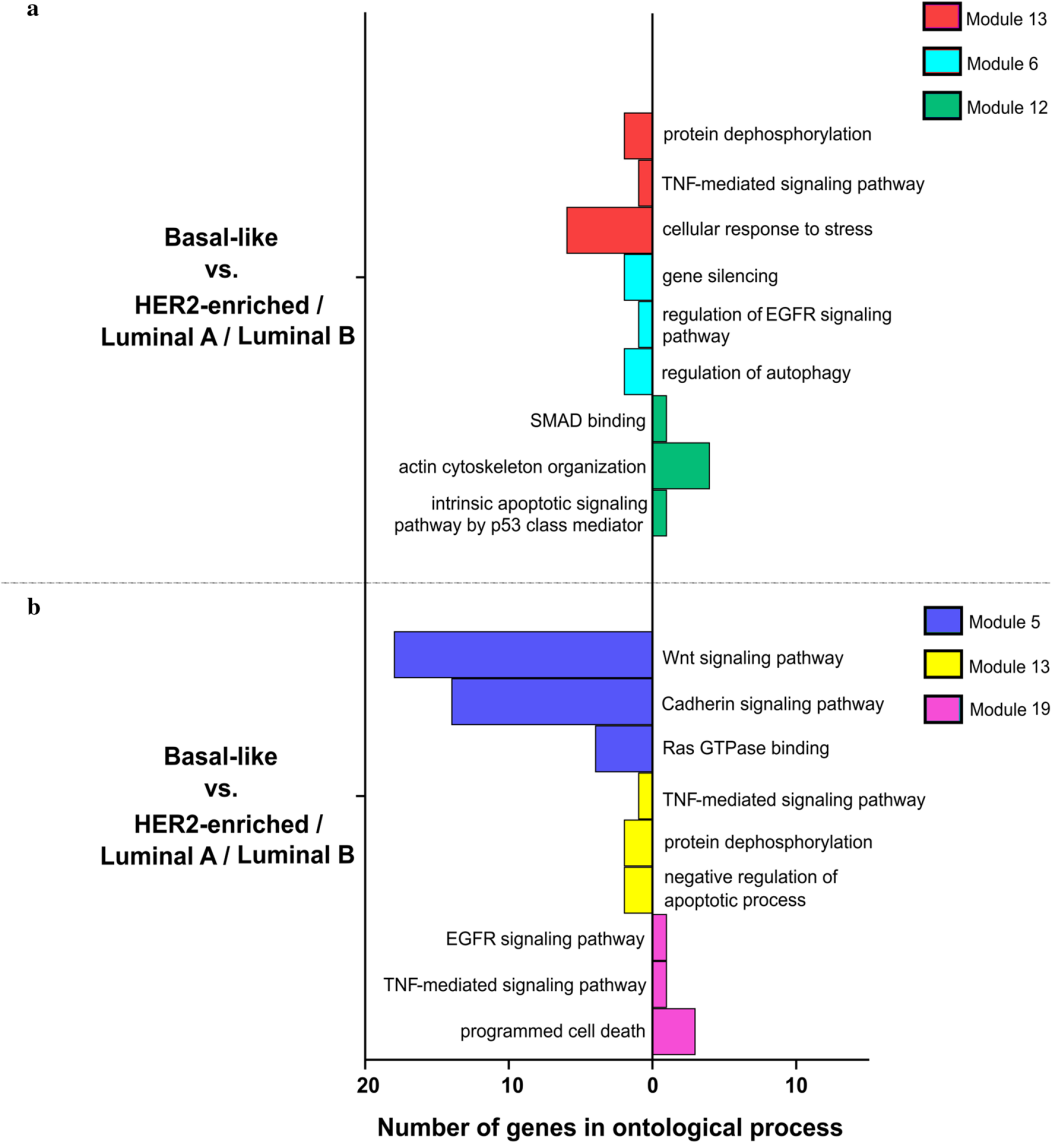
Gene ontology of selected modules differentiating BRCA subtypes by means of AP-2α and AP-2γ targets. **a** Comparisons through AP-2α targets. **b** Comparisons through AP-2γ targets

Pursuing the scheme of previous subsections, gene ontology of modules distinguishing basal-like BRCA signature from other subtypes indicated differences e.g. in apoptosis, cytoskeleton organization, autophagy, but also in EGFR, TNF, Wnt or cadherin signaling pathway. The results are summarized in Fig. 11 and Additional file 4.

### Validation of the Results

Validation of the findings based on independent adequate cohorts for all considered tumors was not performed due to lack of relevant expression data.

## Discussion

Carcinogenesis is shaped by both tumor suppressors and oncogenes where one group of crucial proteins that demonstrate such duality of function are transcription factors. Available literature indicates that the character of AP-2 factors depends on the particular family representative or considered tumor type. Assurance definitely applies to fact that AP-2α and AP-2γ have been described to the greatest extent compared to other family members.

Unlike previous papers, the present study examines the activities of the target genes of AP-2α and AP-2γ in more than 20 solid tumors from the TCGA database. The observed alterations in the expression of transcription factors and their effect on target genes identified processes that can potentially distinguish the tumor from its corresponding normal tissue or from other tumor types.

Regarding the first type of comparisons, a multitude of differences were observed; these allowed the identification of more than one module that could distinguish tumor tissue from corresponding normal tissue, with ontological descriptors concerning both biological processes and molecular pathways. A few modules from AP-2α target genes list explained the differences for several tissues at once: modules 2, 11 and 12 respectively differentiated CESC and UCEC, HNSC and KIRC, as well as COAD, ESCA and STAD from their corresponding normal tissues. Module 2 has been described as implicated in cell cycle regulation as well as the apoptotic pathway, with the cell cycle being differentially regulated between cervical cancer versus solid normal tissue samples [47] or uterine corpus endometrial carcinoma versus normal controls [48]. Following, in module 11 the processes involving e.g. MAPK activity was noticed which could support previous observations that MAPK may play a role in HNSC and KIRC carcinogenesis [49, 50]. Lastly, module 12 was annotated with processes involved in cell cycle or cell adhesion. Differences in cell cycle regulation were previously found between tumor tissue and paired normal samples of colon adenocarcinoma [51], while cell adhesion processes were found to distinguish tumor samples from normal tissue in esophageal carcinoma [52] or gastric cancer [53].

The remaining modules selected for gene ontology analysis indicated differences in PRAD, LUSC, BLCA, GBM, READ, LUAD, BRCA and KIRP between tumors and their matched solid normal tissues. Module 18 shown alterations in apoptosis, the ERBB pathway and regulation of cytoskeleton organization between PRAD and its paired control which is consistent with previous findings for programmed cell death [54] and ERBB signaling [55]. The other pathway shown was Wnt, describing lung squamous cell carcinoma versus normal tissue differences with module 15 which corresponded to literature data on cellular levels of this protein [56]. Furthermore, “regulation of cell cycle” or “response to drug” descriptors of module 13 explained bladder cancer versus normal bladder tissue; this is consistent with current scientific reports where the drug resistance is dependent on the JUND representative of the Jun family [57, 58], which was a part of AP-2α target genes list forming module 13 along with two other Jun members. Module 10 concerns the misfolding of proteins which appeared to be crucial mechanism of lipid storage or increased cellular proliferation and varied between glioblastoma multiforme and corresponding normal tissue [59]. Module 8 revealed differences in intrinsic apoptosis, among others, between rectum adenocarcinoma and controls as noted previously [60]; the same signaling pathway was annotated in module 6 explaining lung adenocarcinoma versus normal lung, although with an additional cell adhesion descriptor which might be related to changes in adhesive properties during epithelial-to-mesenchymal transition, as confirmed via AP-2α–promoted tumorigenicity of LUAD [61]. Finally, kidney renal papillary and breast invasive carcinomas were distinguished from their paired normal tissues using module 1 and 3, respectively. Among the others, signaling pathways such as Wnt, Notch (for KIRP) or ERBB (for BRCA) were implicated in explanation which is coherent with other research [62, 63].

Since the magnitude of the results for AP-2γ is greater than in the case of AP-2α, obtained differences were confronted with the literature only in terms of signaling pathways. Differences in certain pathways, such as TNF or ERBB (for liver hepatocellular carcinoma and prostate adenocarcinoma), EGFR or Wnt (for lung squamous cell carcinoma), JAK-STAT (for kidney renal clear cell carcinoma), NFkB (for breast invasive carcinoma) and TGFβ, mTOR or NFkB (for stomach adenocarcinoma) were noted between tumor and normal tissue. Of these, TNF for LIHC, NFkB for BRCA and TGFβ for STAD have been identified previously [64–66].

Both AP-2α and AP-2γ demonstrated differences associated with signal transduction pathways or biological processes in tumor versus tumor comparisons. Regarding first transcription factor, many of the modules generated through Monocle3 analysis can distinguish KIRC from KIRP; in this, ontological analysis identified changes in processes e.g. cell adhesion, proliferation, angiogenesis, cell migration, cell cycle or pathways i.e. Notch, Wnt, ERBB signaling (Fig. 8). Differences in adhesion or cell cycle have been noted between two renal cell carcinomas, showing specific gene expression to be lower in KIRP [67]. Increased expression of AP-2α targets genes from module 13 (ontologically indicating regulation of cell cycle, proliferation or development) in KIRC compared to KIRP was also noticeable in LGG versus GBM comparison although with inverse tendency (Fig. 8). Considering the cell cycle differences, the alteration frequencies indicate on average higher percentage of pathway disruption in GBM (86%) compared to LGG (46%) [68]. The cell adhesion and intrinsic apoptotic signaling pathway (module 6) remained on trend in both KIRC versus KIRP and LUSC versus LUAD, implying that lung carcinomas differ not only in terms of adhesion or apoptosis but also regarding cell migration or Wnt signaling pathways, as demonstrated by module 15 (Fig. 8). This is consistent with a previous comparison of genes linked to adhesion and migration that were found to be differentially expressed between LUSC and LUAD [69]. Of the two types of lung cancer, LUSC was also found within a mixed cluster (Fig. 2a) created by more similar tumors (LUSC, ESCA, HNSC, UCEC, BLCA, CESC). Nevertheless, the tumors could be distinguished using modules 2, 11 and 19 which included genes related to cell adhesion, regulation of cell cycle arrest and inactivation of MAPK activity.

Concerning differences outlined through AP-2γ targets, identical subgroups could be distinguished within the mixed tumor cluster (Fig. 2b) formed by the same six cancer types described above—distinction is explained by module 17 that post-ontologically shown differences in cell adhesion (Fig. 10), which is concordant with the analysis of the same tumors through AP-2α targets (Fig. 2a). Additionally, module 6 or 15 distinguished two subdivisions inside the cluster i.e. ESCA versus BLCA or UCEC versus LUSC, respectively. Using ontology descriptors, changes concerned regulation of mTOR signaling and extrinsic apoptosis for module 6 or EGFR and Wnt pathways for module 15 (Fig. 10). In regards to the latter, the alteration frequencies indicate on average lower percentage of Wnt pathway disruption in LUSC (18%) compared to UCEC (47%) [68]. Other comparisons applied to COAD versus READ explained by vesicle budding (module 9) or LGG versus GBM elucidated through mTOR signaling, apoptosis and cellular adhesion (module 5) with the latter confirmed by another bioinformatics analysis [70]. Lastly, numerous modules distinguished KIRC from KIRP or LUSC from LUAD with module 12 (response to hypoxia, regulation of autophagy, adhesion-dependent cell spreading) differentiating both. Referring the literature, renal cell carcinomas have already been differentiated in terms of hypoxia indicating specific gene expression to be higher in KIRC [67]. Other modules for KIRC versus KIRP comparison have been associated with processes such as autophagy (module 3), hormone-mediated signaling pathway (module 4) and cell growth regulation or NFkB signaling (module 16). For LUSC versus LUAD, they differ according to genes associated with Wnt pathway (module 10), regulation of cytoskeleton organization (module 7) or EGFR signaling (module 15). The last mentioned might be supported by a threefold difference in the frequency of *EGFR* mutations between LUSC and LUAD during a previous pan-cancer analysis [71].

A separate topic was the division of breast cancer subtypes noticed during the global analysis of all cancer cohorts in relation to their corresponding normal tissues. Both target gene lists i.e. for AP-2α and AP-2γ, have separated two subgroups of BRCA samples. Additional spatial analysis and assignment of PAM50 signatures to samples revealed that the distinguished group was a basal-like subtype (Fig. 4). Due to the fact that this subtype is the most aggressive form of breast cancer [72], finding potential differences from the others could support clinical aspects in the future. Ontological analysis was performed on modules whose constituent genes demonstrated opposite expression profiles (basal-like vs. luminal A/B or HER2-enriched). Mutual part indicated through target genes lists of both AP-2α and AP-2γ shown that the processes differentiating the basal-like subtype concern regulation of programmed cell death or EGFR and TNF signaling pathways (Fig. 11), with the latter observed during previous enrichment analysis [72]. Nevertheless, the cadherin and Wnt signaling pathways play a key role in subtype differentiation due to the number of genes described ontologically in module 5 (for AP-2γ targets’ comparisons), as noted previously [73].

On the whole, the analysis presented in this research indicate that many differentiating processes or pathways between tumor and normal tissue or between different tumor types/subtypes find their reference in current scientific reports. However, some of the obtained alterations not having regard in the literature, could serve as a novel preliminary point for the development of new anti-cancer therapies.

## Conclusions

Collectively, our research indicates both novel and previously described differences between tumors or between a specific tumor and normal matched solid tissue. In addition, an unplanned division of samples from the breast cancer cohort was performed, differentiating the basal-like subtype from the others. These findings could have the clinical applications.

Analyses also proved that the regulation of gene expression by AP-2α and AP-2γ is very complex and involves many biological processes and signaling pathways. The role of AP-2 in carcinogenesis is ambiguous, but it is associated with the regulation of processes that underlie cancer hallmarks (proliferation, apoptosis, angiogenesis, cell growth), and the target genes of both transcription factors allow for further analysis of changes that occur in individual tumors. Finally, the differences in AP-2α and AP-2γ expression observed between tissue types (e.g. THCA and SKCM or KIRC/KIRP vs. normal kidney tissue) suggest their potential usefulness in diagnosing and treating specific cancers.

Nonetheless, we believe that our findings regarding AP-2α and AP-2γ functional genomics sheds new light on specific comparisons yet it is entirely possible that subsequent discoveries through in-depth data mining could have a further impact on the future of medical practice.

## Supplementary information

**Additional file 1**. Content of modules from particular Monocle3 approach.

**Additional file 2**. Detailed ontological analysis of selected modules differentiating tumor and corresponding normal tissue by means of AP-2α target genes.

**Additional file 3**. Detailed ontological analysis of selected modules differentiating tumor and corresponding normal tissue by means of AP-2γ target genes.

**Additional file 4**. Detailed ontological analysis of selected modules differentiating basal-like breast cancer from other subtypes by means of AP-2α and AP-2γ lists of target genes.

## Data Availability

TCGA gene expression profiles (level 3 RNA-seqV2, RSEM normalized) are publicly available in the Broad GDAC Firehose repository (https://gdac.broadinstitute.org/) wherein cohort abbreviations used in the present study are accession identifiers (data status of 28th Jan 2016). Direct access to respective cohort is possible through http://firebrowse.org/?cohort=X where “X” should be changed to cohort abbreviation (e.g. BLCA, BRCA, CESC, etc.).

## Abbreviations

TFs: Transcription factors
AP-2: Activating enhancer-binding Protein 2
WWOX: WW Domain Containing Oxidoreductase
bHSH: Basic Helix–Span–Helix
ERα: Estrogen receptor alpha
TCGA: The Cancer Genome Atlas
PANTHER: Protein Analysis Through Evolutionary Relationships
BLCA: Bladder urothelial carcinoma
BRCA: Breast invasive carcinoma
CESC: Cervical and endocervical cancers
COAD: Colon adenocarcinoma
ESCA: Esophageal carcinoma
GBM: Glioblastoma multiforme
HNSC: Head and neck squamous cell carcinoma
KIRC: Kidney renal clear cell carcinoma
KIRP: Kidney renal papillary cell carcinoma
LGG: Brain lower grade glioma
LIHC: Liver hepatocellular carcinoma
LUAD: Lung adenocarcinoma
LUSC: Lung squamous cell carcinoma
OV: Ovarian serous cystadenocarcinoma
PAAD: Pancreatic adenocarcinoma
PRAD: Prostate adenocarcinoma
READ: Rectum adenocarcinoma
SKCM: Skin cutaneous melanoma
STAD: Stomach adenocarcinoma
THCA: Thyroid carcinoma
UCEC: Uterine corpus endometrial carcinoma
UMAP: Uniform Manifold Approximation and Projection
PAM50: Prosigna Breast Cancer Prognostic Gene Signature Assay
GTRD: Gene Transcription Regulation Database
TRANSFAC: TRANScription FACtor database
TRRUST: Transcriptional Regulatory Relationships Unraveled by Sentence-based Text mining
PCA: Principle Component Analysis

## References

[1] Nagai H, Kim YH (2017). Cancer prevention from the perspective of global cancer burden patterns. J Thorac Dis.

[2] Hyndman IJ (2016). Review: the contribution of both nature and nurture to carcinogenesis and progression in solid tumours. Cancer Microenviron.

[3] Jilkine A, Gutenkunst RN (2014). Effect of dedifferentiation on time to mutation acquisition in stem cell-driven cancers. PLoS Comput Biol.

[4] Cortessis VK, Thomas DC, Levine AJ, Breton CV, Mack TM, Siegmund KD (2012). Environmental epigenetics: prospects for studying epigenetic mediation of exposure-response relationships. Hum Genet.

[5] Paduch R (2015). Theories of cancer origin. Eur J Cancer Prev.

[6] Sonnenschein C, Soto AM (2016). Carcinogenesis explained within the context of a theory of organisms. Prog Biophys Mol Biol.

[7] Zhu K, Liu Q, Zhou Y, Tao C, Zhao Z, Sun J (2015). Oncogenes and tumor suppressor genes: comparative genomics and network perspectives. BMC Genom.

[8] Pedraza-Farina LG (2006). Mechanisms of oncogenic cooperation in cancer initiation and metastasis. Yale J Biol Med.

[9] Lou X, Zhang J, Liu S, Xu N, Liao DJ (2014). The other side of the coin: the tumor-suppressive aspect of oncogenes and the oncogenic aspect of tumor-suppressive genes, such as those along the CCND-CDK4/6-RB axis. Cell Cycle.

[10] Muller PA, Vousden KH (2014). Mutant p53 in cancer: new functions and therapeutic opportunities. Cancer Cell.

[11] Ruiz M, Pettaway C, Song R, Stoeltzing O, Ellis L, Bar-Eli M (2004). Activator protein 2alpha inhibits tumorigenicity and represses vascular endothelial growth factor transcription in prostate cancer cells. Cancer Res.

[12] Shi D, Xie F, Zhang Y, Tian Y, Chen W, Fu L (2014). TFAP2A regulates nasopharyngeal carcinoma growth and survival by targeting HIF-1alpha signaling pathway. Cancer Prev Res (Phila).

[13] Aqeilan RI, Palamarchuk A, Weigel RJ, Herrero JJ, Pekarsky Y, Croce CM (2004). Physical and functional interactions between the Wwox tumor suppressor protein and the AP-2gamma transcription factor. Cancer Res.

[14] Li H, Goswami PC, Domann FE (2006). AP-2gamma induces p21 expression, arrests cell cycle, and inhibits the tumor growth of human carcinoma cells. Neoplasia.

[15] Kolat D, Kaluzinska Z, Bednarek AK, Pluciennik E (2019). The biological characteristics of transcription factors AP-2alpha and AP-2gamma and their importance in various types of cancers. Biosci Rep.

[16] Eckert D, Buhl S, Weber S, Jager R, Schorle H (2005). The AP-2 family of transcription factors. Genome Biol.

[17] Mohibullah N, Donner A, Ippolito JA, Williams T (1999). SELEX and missing phosphate contact analyses reveal flexibility within the AP-2[alpha] protein: DNA binding complex. Nucleic Acids Res.

[18] Mitchell PJ, Wang C, Tjian R (1987). Positive and negative regulation of transcription in vitro: enhancer-binding protein AP-2 is inhibited by SV40 T antigen. Cell.

[19] Hilger-Eversheim K, Moser M, Schorle H, Buettner R (2000). Regulatory roles of AP-2 transcription factors in vertebrate development, apoptosis and cell-cycle control. Gene.

[20] Wu HR, Zhang J (2018). AP-2alpha expression in papillary thyroid carcinoma predicts tumor progression and poor prognosis. Cancer Manag Res.

[21] Anttila MA, Kellokoski JK, Moisio KI, Mitchell PJ, Saarikoski S, Syrjanen K (2000). Expression of transcription factor AP-2alpha predicts survival in epithelial ovarian cancer. Br J Cancer.

[22] Wang W, Lv L, Pan K, Zhang Y, Zhao JJ, Chen JG (2011). Reduced expression of transcription factor AP-2alpha is associated with gastric adenocarcinoma prognosis. PLoS ONE.

[23] Perkins SM, Bales C, Vladislav T, Althouse S, Miller KD, Sandusky G (2015). TFAP2C expression in breast cancer: correlation with overall survival beyond 10 years of initial diagnosis. Breast Cancer Res Treat.

[24] Gee JM, Eloranta JJ, Ibbitt JC, Robertson JF, Ellis IO, Williams T (2009). Overexpression of TFAP2C in invasive breast cancer correlates with a poorer response to anti-hormone therapy and reduced patient survival. J Pathol.

[25] Wang X, Sun D, Tai J, Chen S, Yu M, Ren D (2018). TFAP2C promotes stemness and chemotherapeutic resistance in colorectal cancer via inactivating hippo signaling pathway. J Exp Clin Cancer Res.

[26] Lin CY, Chao A, Wang TH, Lee LY, Yang LY, Tsai CL (2016). Nucleophosmin/B23 is a negative regulator of estrogen receptor alpha expression via AP2gamma in endometrial cancer cells. Oncotarget.

[27] Zhang P, Hou Q, Yue Q (2020). MiR-204-5p/TFAP2A feedback loop positively regulates the proliferation, migration, invasion and EMT process in cervical cancer. Cancer Biomark.

[28] Huang HX, Yang G, Yang Y, Yan J, Tang XY, Pan Q (2020). TFAP2A is a novel regulator that modulates ferroptosis in gallbladder carcinoma cells via the Nrf2 signalling axis. Eur Rev Med Pharmacol Sci.

[29] Sliwa A, Kubiczak M, Szczerba A, Walkowiak G, Nowak-Markwitz E, Burczynska B (2019). Regulation of human chorionic gonadotropin beta subunit expression in ovarian cancer. BMC Cancer.

[30] Pellikainen J, Kataja V, Ropponen K, Kellokoski J, Pietilainen T, Bohm J (2002). Reduced nuclear expression of transcription factor AP-2 associates with aggressive breast cancer. Clin Cancer Res.

[31] Su W, Xia J, Chen X, Xu M, Nie L, Chen N (2014). Ectopic expression of AP-2alpha transcription factor suppresses glioma progression. Int J Clin Exp Pathol.

[32] Hallberg AR, Vorrink SU, Hudachek DR, Cramer-Morales K, Milhem MM, Cornell RA (2014). Aberrant CpG methylation of the TFAP2A gene constitutes a mechanism for loss of TFAP2A expression in human metastatic melanoma. Epigenetics.

[33] Lian W, Zhang L, Yang L, Chen W (2017). AP-2alpha reverses vincristine-induced multidrug resistance of SGC7901 gastric cancer cells by inhibiting the Notch pathway. Apoptosis.

[34] Makhov PB, Golovine KV, Kutikov A, Canter DJ, Rybko VA, Roshchin DA (2011). Reversal of epigenetic silencing of AP-2alpha results in increased zinc uptake in DU-145 and LNCaP prostate cancer cells. Carcinogenesis.

[35] Li Q, Dashwood RH (2004). Activator protein 2alpha associates with adenomatous polyposis coli/beta-catenin and Inhibits beta-catenin/T-cell factor transcriptional activity in colorectal cancer cells. J Biol Chem.

[36] Hu J, Tan SK, Lim MGL, Chang SH, Cui G, Liu S (2018). Identification of a Wells-Dawson polyoxometalate-based AP-2gamma inhibitor with pro-apoptotic activity. Biochem J.

[37] Li Z, Xu X, Luo M, Hao J, Zhao S, Yu W (2018). Activator protein-2beta promotes tumor growth and predicts poor prognosis in breast cancer. Cell Physiol Biochem.

[38] Fu X, Zhang H, Chen Z, Yang Z, Shi D, Liu T (2019). TFAP2B overexpression contributes to tumor growth and progression of thyroid cancer through the COX-2 signaling pathway. Cell Death Dis.

[39] Fraune C, Harms L, Buscheck F, Hoflmayer D, Tsourlakis MC, Clauditz TS (2020). Upregulation of the transcription factor TFAP2D is associated with aggressive tumor phenotype in prostate cancer lacking the TMPRSS2:ERG fusion. Mol Med.

[40] Hoshi R, Watanabe Y, Ishizuka Y, Hirano T, Nagasaki-Maeoka E, Yoshizawa S (2017). Depletion of TFAP2E attenuates adriamycin-mediated apoptosis in human neuroblastoma cells. Oncol Rep.

[41] Wei L, Jin Z, Yang S, Xu Y, Zhu Y, Ji Y (2018). TCGA-assembler 2: software pipeline for retrieval and processing of TCGA/CPTAC data. Bioinformatics.

[42] Yevshin I, Sharipov R, Valeev T, Kel A, Kolpakov F (2017). GTRD: a database of transcription factor binding sites identified by ChIP-seq experiments. Nucleic Acids Res.

[43] Yevshin I, Sharipov R, Kolmykov S, Kondrakhin Y, Kolpakov F (2019). GTRD: a database on gene transcription regulation-2019 update. Nucleic Acids Res.

[44] Trapnell C, Cacchiarelli D, Grimsby J, Pokharel P, Li S, Morse M (2014). The dynamics and regulators of cell fate decisions are revealed by pseudotemporal ordering of single cells. Nat Biotechnol.

[45] Qin S, Shi X, Wang C, Jin P, Ma F (2019). Transcription factor and miRNA interplays can manifest the survival of ccRCC patients. Cancers (Basel).

[46] Lommen K, Vaes N, Aarts MJ, van Roermund JG, Schouten LJ, Oosterwijk E (2019). Diagnostic DNA methylation biomarkers for renal cell carcinoma: a systematic review. Eur Urol Oncol.

[47] Chen P, Zhang W, Chen Y, Zheng X, Yang D (2020). Comprehensive analysis of aberrantly expressed long noncoding RNAs, microRNAs, and mRNAs associated with the competitive endogenous RNA network in cervical cancer. Mol Med Rep.

[48] Shen L, Liu M, Liu W, Cui J, Li C (2018). Bioinformatics analysis of RNA sequencing data reveals multiple key genes in uterine corpus endometrial carcinoma. Oncol Lett.

[49] Li ZX, Zheng ZQ, Wei ZH, Zhang LL, Li F, Lin L (2019). Comprehensive characterization of the alternative splicing landscape in head and neck squamous cell carcinoma reveals novel events associated with tumorigenesis and the immune microenvironment. Theranostics.

[50] Huang D, Ding Y, Luo WM, Bender S, Qian CN, Kort E (2008). Inhibition of MAPK kinase signaling pathways suppressed renal cell carcinoma growth and angiogenesis in vivo. Cancer Res.

[51] Hu M, Fu X, Si Z, Li C, Sun J, Du X (2019). Identification of differently expressed genes associated with prognosis and growth in colon adenocarcinoma based on integrated bioinformatics analysis. Front Genet.

[52] Zeng JH, Xiong DD, Pang YY, Zhang Y, Tang RX, Luo DZ (2017). Identification of molecular targets for esophageal carcinoma diagnosis using miRNA-seq and RNA-seq data from The Cancer Genome Atlas: a study of 187 cases. Oncotarget.

[53] Marimuthu A, Jacob HK, Jakharia A, Subbannayya Y, Keerthikumar S, Kashyap MK (2011). Gene expression profiling of gastric cancer. J Proteomics Bioinform.

[54] Kulik G (2015). Personalized prostate cancer therapy based on systems analysis of the apoptosis regulatory network. Asian J Androl.

[55] Miller DR, Ingersoll MA, Lin MF (2019). ErbB-2 signaling in advanced prostate cancer progression and potential therapy. Endocr Relat Cancer.

[56] Bartis D, Csongei V, Weich A, Kiss E, Barko S, Kovacs T (2013). Down-regulation of canonical and up-regulation of non-canonical Wnt signalling in the carcinogenic process of squamous cell lung carcinoma. PLoS ONE.

[57] Mitra AP, Hansel DE, Cote RJ (2012). Prognostic value of cell-cycle regulation biomarkers in bladder cancer. Semin Oncol.

[58] Peng Y, Chen Y, Chen S, Wang J, Jiang C, Hou W (2020). JUND-dependent up-regulation of HMOX1 is associated with cisplatin resistance in muscle-invasive bladder cancer. J Biochem.

[59] Madden E, Logue SE, Healy SJ, Manie S, Samali A (2019). The role of the unfolded protein response in cancer progression: From oncogenesis to chemoresistance. Biol Cell.

[60] Benard A, Zeestraten EC, Goossens-Beumer IJ, Putter H, van de Velde CJ, Hoon DS (2014). DNA methylation of apoptosis genes in rectal cancer predicts patient survival and tumor recurrence. Apoptosis.

[61] Yuanhua L, Pudong Q, Wei Z, Yuan W, Delin L, Yan Z (2019). TFAP2A induced KRT16 as an oncogene in lung adenocarcinoma via EMT. Int J Biol Sci.

[62] Fendler A, Bauer D, Busch J, Jung K, Wulf-Goldenberg A, Kunz S (2020). Inhibiting WNT and NOTCH in renal cancer stem cells and the implications for human patients. Nat Commun.

[63] Zghair AN, Sinha DK, Kassim A, Alfaham M, Sharma AK (2016). Differential Gene Expression of BRCA1, ERBB2 and TP53 biomarkers between human breast tissue and peripheral blood samples of breast cancer. Anticancer Agents Med Chem.

[64] Tan W, Luo X, Li W, Zhong J, Cao J, Zhu S (2019). TNF-alpha is a potential therapeutic target to overcome sorafenib resistance in hepatocellular carcinoma. EBioMedicine.

[65] Sau A, Cabrita MA, Pratt MAC (2018). NF-kappaB at the crossroads of normal mammary gland biology and the pathogenesis and prevention of BRCA1-mutated breast cancer. Cancer Prev Res (Phila).

[66] Ishimoto T, Miyake K, Nandi T, Yashiro M, Onishi N, Huang KK (2017). Activation of transforming growth factor beta 1 signaling in gastric cancer-associated fibroblasts increases their motility, via expression of rhomboid 5 homolog 2, and ability to induce invasiveness of gastric cancer cells. Gastroenterology.

[67] Chen F, Zhang Y, Senbabaoglu Y, Ciriello G, Yang L, Reznik E (2016). Multilevel genomics-based taxonomy of renal cell carcinoma. Cell Rep.

[68] Sanchez-Vega F, Mina M, Armenia J, Chatila WK, Luna A, La KC (2018). Oncogenic signaling pathways in the cancer genome atlas. Cell.

[69] Chen M, Liu X, Du J, Wang XJ, Xia L (2017). Differentiated regulation of immune-response related genes between LUAD and LUSC subtypes of lung cancers. Oncotarget.

[70] Xu Y, Geng R, Yuan F, Sun Q, Liu B, Chen Q (2019). Identification of differentially expressed key genes between glioblastoma and low-grade glioma by bioinformatics analysis. PeerJ.

[71] Liu H, Zhang B, Sun Z (2020). Spectrum of EGFR aberrations and potential clinical implications: insights from integrative pan-cancer analysis. Cancer Commun (Lond).

[72] Yang K, Gao J, Luo M (2019). Identification of key pathways and hub genes in basal-like breast cancer using bioinformatics analysis. Onco Targets Ther.

[73] Jiang S, Zhang M, Zhang Y, Zhou W, Zhu T, Ruan Q (2019). WNT5B governs the phenotype of basal-like breast cancer by activating WNT signaling. Cell Commun Signal.

