# Supplementary material for: Functional genomics of AP-2α and AP-2γ in cancers: in silico study
Source: BMC Med Genomics. 2020 Nov 19;13:174. doi: 10.1186/s12920-020-00823-9 (PMC7678100; doi:10.1186/s12920-020-00823-9)
Supplement: Supplementary file 2 — Additional file 2. Detailed ontological analysis of selected modules differentiating tumor and corresponding normal tissue by means of AP-2α target genes. [file 12920_2020_823_MOESM2_ESM.docx]

**Additional file 2. Detailed ontological analysis of selected modules differentiating tumor and corresponding normal tissue by means of AP-2α target genes.**

| **Module** | **Biological process** | **Genes** | **p-value** |
| --- | --- | --- | --- |
| 1 | negative regulation of extrinsic apoptotic signaling pathway | LGALS3  EYA4  EYA2  EYA1 | 3.00E-02 |
|  | negative regulation of extrinsic apoptotic signaling pathway | SEMA3B  SEMA4B  PLCG2  SEMA4D  SEMA4C  PDGFA  SEMA5A  SEMA3F  MADCAM1  VEGFA  SEMA5B  SEMA3E  PDGFC  PDGFRB  SEMA6A  SEMA3C  SEMA6D  PGF  SEMA7A  SEMA3D  SEMA3A  KIT | 1.84E-05 |
|  | angiogenesis | GATA2  ACVRL1  VASH1  COL22A1  ACVR1  AMOT  AMOTL1  COL15A1  VEGFA  ISM1  RAMP1  PGF  COL18A1  VASH2  TBX4 | 2.81E-03 |
|  | positive regulation of MAPK cascade | ARRB2  TNFRSF19  MAP3K9  ARRB1  IL11  PDE8A  INSR  CX3CL1  TRAF4  MAP2K5  IGF1R  DUSP15  PDGFA  EDAR  CD4  GADD45G  PDGFC  TRAF5  FGFR3  INSRR  SEMA7A  MAP3K12  MAP3K13  RELL1  TRAF1 KIT | 9.58E-03 |
|  | positive regulation of Wnt signaling pathway | GPC3  LYPD6  GPC5  PPM1N  PPM1B  SULF2  CSNK1G1  SULF1  FAM53B | 1.65E-02 |
|  | Notch signaling pathway | LLGL2  DTX1  DLL1  ARRB1  DLG4  AAK1  RBPJ  MAML3  MIB1  LFNG  PEAR1  NOTCH3  MAML2 | 2.46E-02 |
|  | cell population proliferation | KITLG  TGFB1  TFAP2B  STAT2  IL27RA  PTK6  TGFA  IL11  STAT4  ERBB3  BCL6  PDCD1LG2  TFAP2A  STAT5A  DUSP15  ALK  EGFR  TMEM119  PDGFA  TGFB3  LYN  FYN  TNFRSF13C  STAT1  VEGFA  CA11  TBX3  SRMS  ZAP70  PDGFC  SLA  ERBB4  TFAP2E  FER  PGF  IL34  IL6R | 8.70E-03 |
| 2 | regulation of cell cycle arrest | ARID3A  BRCA1  E2F1  HSP90AB1  CARM1  ZBTB17  CRADD  TP73  AKT2  FOXM1  PRMT1 | 8.99E-03 |
|  | negative regulation of cell cycle | ARID3A  CTDSP2  BRCA1  BRIP1  E2F1  PTTG1  EHMT2  HSP90AB1  CDKN2C  CLSPN  CARM1  ZBTB17  RAD51  CRADD  PSMD9  TP73  CHFR  STK11  CDC14A  CDT1  EZH2  TAOK3  DOT1L  MAPK14  CDC6  CCNF  PRMT1 | 4.16E-02 |
|  | regulation of G1/S transition of mitotic cell cycle | ARID3A  CTDSP2  E2F1  CDKN2C  CARM1  CRADD  TCF3  CCND3  EZH2  APPL2  CDC6  PRMT1 | 3.03E-02 |
|  | apoptotic signaling pathway | MKNK2  BRCA1  E2F1  ACVR1B  E2F2  HIPK1  TMEM117  SOD2  CRADD  UBE4B  TP73  STK11  BCL3  PPARD  TIMM50  DEDD2  TMBIM6  TNFRSF1A | 1.69E-02 |
| 3 | Ras protein signal transduction | ARHGDIA  ARHGAP17  RAB5C  GNA13  CDC42EP4 | 1.04E-02 |
|  | ERBB signaling pathway | RHBDF2  RHBDF1 | 2.03E-02 |
| 4 | regulation of mitotic cell cycle | CDK10  CDC25B  CDC14B  RBL2 | 1.96E-02 |
|  | positive regulation of stress-activated MAPK cascade | TRAF2  MAPK8IP3 | 2.40E-02 |
|  | protein polyubiquitination | TRAF2  WWP2  AMFR | 4.27E-02 |
| 6 | cell adhesion | PCDHA6  PCDHA9  PCDHA2  PCDHA4  PCDHA11  PCDHA7  PCDHAC2  PCDHA13  PCDHA10  PCDHA3  PCDHA8  PCDHA12  PCDHA5  PCDHA1  PCDHAC1 | 1.05E-12 |
|  | intrinsic apoptotic signaling pathway in response to DNA damage by p53 class mediator | HIPK2 | 2.30E-02 |
| 8 | regulation of intrinsic apoptotic signaling pathway | BCL2L1 | 4.27E-02 |
|  | protein polyubiquitination | UBE2V1  RBCK1 | 4.16E-02 |
| 9 | positive regulation of programmed cell death | BID  PPM1F | 1.44E-02 |
|  | regulation of cell shape | CDC42EP1  PLXNB2 | 1.23E-02 |
| 10 | cellular response to misfolded protein | DNAJB12 | 4.11E-02 |
|  | regulation of gene expression | BMPR1A  ARID5B  TCF7L2  MAPK8  SUFU  ZMIZ1  CPEB3  TET1  JMJD1C  LDB1 | 2.57E-02 |
| 11 | protein ubiquitination | ARIH2  RAD18  UBE2E1 | 1.84E-02 |
|  | inactivation of MAPK activity | DUSP7 | 4.52E-02 |
|  | intracellular signal transduction | LIMD1  ARHGEF3  DUSP7  PRKCD  PLCD1  SNRK  MAPKAPK3  GNAI2 | 5.00E-03 |
| 12 | negative regulation of G2/M transition of mitotic cell cycle | NBN | 3.61E-02 |
|  | regulation of cell adhesion | FAM49B  PTK2 | 1.17E-02 |
|  | mitotic nuclear division | NBN  STK3  KIFC2 | 4.50E-02 |
| 13 | regulation of cell cycle | JUN  JUNB  JUND  PLK3  WEE1 | 4.14E-04 |
|  | mitotic cell cycle checkpoint | PLK3  WEE1 | 5.51E-03 |
|  | response to drug | JUN  JUNB  JUND | 3.36E-03 |
|  | regulation of cell population proliferation | JUN  JUNB  JUND | 6.79E-03 |
|  | regulation of cell development | HES1  BHLHE40 | 4.38E-02 |
| 15 | canonical Wnt signaling pathway | GSK3B  DVL3 | 1.59E-02 |
|  | cell migration | ITGB5  AMOTL2  PLXNA1  PIK3CB | 4.36E-03 |
|  | regulation of cell morphogenesis involved in differentiation | PLXNA1  PAK2 | 8.30E-03 |
| 16 | cell cycle | MINK1  RNF167  LLGL1  NDEL1 | 3.34E-02 |
|  | regulation of Notch signaling pathway | LLGL1 | 4.71E-02 |
| 17 | cell cycle process | LATS2  RB1  TUBGCP3  PDS5B | 1.10E-02 |
|  | G1/S transition of mitotic cell cycle | LATS2  RB1 | 1.15E-03 |
|  | negative regulation of cell migration | LRCH1 | 2.78E-02 |
|  | drug transmembrane transport | SLC7A1 | 3.56E-02 |
| 18 | apoptotic process | XKR6  BNIP3L | 4.58E-02 |
|  | ERBB signaling pathway | PTK2B | 3.83E-02 |
|  | regulation of cytoskeleton organization | RHOBTB2  ARHGEF10 | 2.97E-02 |
| 19 | cell adhesion | PCDHGB2  PCDHGB3  PCDHGA3  PCDHGB7  PCDHGA11  PCDHGA5  PCDHGA12  PCDHGC4  PCDHGA8  PCDHGA10  PCDHGB1  PCDHGA6  PCDHGC3  PCDHGB4  PCDHGA4  PCDHGB6  PCDHGB5  PCDHGA2  PCDHGA1  PCDHGA7  PCDHGA9  PCDHGC5 | 1.68E-38 |
