# Supplementary material for: Functional genomics of AP-2α and AP-2γ in cancers: in silico study
Source: BMC Med Genomics. 2020 Nov 19;13:174. doi: 10.1186/s12920-020-00823-9 (PMC7678100; doi:10.1186/s12920-020-00823-9)
Supplement: Supplementary file 3 — Additional file 3. Detailed ontological analysis of selected modules differentiating tumor and corresponding normal tissue by means of AP-2γ target genes. [file 12920_2020_823_MOESM3_ESM.docx]

**Additional file 3. Detailed ontological analysis of selected modules differentiating tumor and corresponding normal tissue by means of AP-2γ target genes.**

| **Module** | **Biological process** | **Genes** | **p-value** |
| --- | --- | --- | --- |
| 1 | extracellular matrix organization | MMP17  NTNG2  MMP28  LOXL1  LOXL4  LAMB4  LAMB1  COL11A2  FBLN5  NTN4  SULF2  MMP16  HAS2  COL2A1  COL13A1  LOXL2  SULF1  NTNG1  TGFBI  MMP11  LOXL3  COL5A1  MMP2 | 9.91E-05 |
|  | negative regulation of autophagy | DAPL1  BMF  DAP  MTOR | 2.96E-02 |
|  | negative regulation of extrinsic apoptotic signaling pathway | LGALS3  EYA4  EYA2  EYA1 | 2.96E-02 |
|  | regulation of cell growth | SEMA3B  SEMA4D  SEMA4C  SEMA5A  SEMA3F  WFDC1  SEMA5B  DBNL  SEMA3E  SEMA6A  SEMA3C  OSGIN1  SEMA6D  SEMA7A  SEMA3A | 1.27E-04 |
|  | negative regulation of MAP kinase activity | DUSP6  DUSP1  DUSP10  PTPRJ  SPRED2  DUSP8  DUSP4 | 4.04E-02 |
| 2 | NIK/NF-kappaB signaling | REL  RELA | 4.50E-02 |
|  | transforming growth factor beta receptor signaling pathway | ACVR1  PXN  BCL9L | 4.50E-02 |
|  | substrate adhesion-dependent cell spreading | PARVA  PXN  LIMS1 | 2.75E-02 |
| 3 | autophagy | CLEC16A  MAP1LC3B  ATG16L2  NPRL3 | 4.23E-02 |
|  | protein ubiquitination | SMURF1  WWP2  UBE3C  AMFR | 2.82E-02 |
| 4 | positive regulation of cell growth | RPTOR | 3.57E-02 |
|  | hormone-mediated signaling pathway | RARA  THRA | 3.55E-02 |
|  | proteasome-mediated ubiquitin-dependent protein catabolic process | SMURF2  TNFAIP1  SPOP  KCTD2  FBXL20 | 1.30E-02 |
|  | positive regulation of protein serine/threonine kinase activity | SPAG9  RPTOR | 4.73E-02 |
| 5 | release of cytochrome c from mitochondria | BID | 2.10E-02 |
|  | positive regulation of cysteine-type endopeptidase activity involved in apoptotic process | BID  PPM1F | 7.11E-03 |
|  | cell adhesion | PCDHA6  PCDHA9  PCDHA2  PCDHA4  PCDHA11  PCDHA7  PCDHA13  PCDHA10  PCDHA3  PCDHA8  PCDHA12  PCDHA5  PCDHA1  ARVCF  PARVB  PLXNB2  PCDHAC1 | 8.86E-13 |
|  | TOR signaling | PRR5  DEPDC5 | 2.18E-02 |
| 6 | negative regulation of TOR signaling | GSK3A  AKT1S1 | 4.99E-03 |
|  | extrinsic apoptotic signaling pathway | GSK3A  DEDD2 | 1.17E-02 |
|  | TOR signaling | GSK3A  AKT1S1 | 4.99E-03 |
| 7 | regulation of exit from mitosis | CDC14B | 2.41E-02 |
|  | chromatin remodeling | SMARCA2  KDM4C | 3.57E-02 |
|  | small molecule catabolic process | SLC27A4  GALT  AUH | 1.91E-02 |
|  | regulation of microtubule cytoskeleton organization | BICD2  CAMSAP1 | 1.70E-02 |
| 8 | negative regulation of I-kappaB kinase/NF-kappaB signaling | OTUD7B | 3.07E-02 |
|  | negative regulation of cell migration | SRGAP2 | 1.97E-03 |
|  | neuron migration | SDCCAG8 | 3.07E-02 |
| 9 | vesicle budding from membrane | SNX5  ATP9A  ARFGAP1  ARFGAP1 | 1.88E-02 |
|  | regulation of cellular macromolecule biosynthetic process | ZHX3  ZNF341  GMEB2  SS18L1  TCFL5  NCOA3  CBFA2T2  NECAB3  TAF4  NCOA6 | 4.38E-02 |
| 10 | protein quality control for misfolded or incompletely synthesized proteins | LONP1 | 4.93E-02 |
|  | canonical Wnt signaling pathway | TCF3  CSNK1G2 | 4.07E-02 |
|  | neurogenesis | BSG  TCF3  BTBD2  ZSWIM4 | 4.34E-02 |
| 12 | protein polyubiquitination | ARIH2  UBE2E1 | 4.46E-02 |
|  | positive regulation of autophagy | FYCO1 | 3.35E-02 |
|  | response to growth factor | TGFBR2  ACVR2B | 4.83E-02 |
|  | substrate adhesion-dependent cell spreading | LAMB2 | 3.68E-03 |
|  | response to hypoxia | LIMD1 | 4.80E-02 |
| 15 | canonical Wnt signaling pathway | GSK3B  DVL3 | 2.61E-02 |
|  | NLS-bearing protein import into nucleus | KPNA1 | 3.25E-02 |
|  | regulation of epidermal growth factor receptor signaling pathway | CBLB | 3.89E-02 |
| 16 | regulation of cell growth | CDKL3  DBN1 | 4.11E-03 |
|  | negative regulation of I-kappaB kinase/NF-kappaB signaling | TNIP1 | 2.29E-02 |
|  | regulation of cell size | CDKL3  DBN1 | 5.95E-03 |
| 17 | cell adhesion | PCDHGB2  PCDHGB3  PCDHGA3  PCDHGA5  PCDHGA8  PCDHGB1  PCDHGA6  PCDHGB4  PCDHGA4  PCDHGB6  PCDHGB5  PCDHGA2  PCDHGA1  PCDHGA7  PCDHGA9 | 3.35E-14 |
| 19 | apoptotic process | TNFRSF10B  XKR6  BNIP3L | 8.40E-03 |
|  | tumor necrosis factor-mediated signaling pathway | IKBKB | 2.76E-02 |
|  | ERBB signaling pathway | PTK2B | 4.63E-02 |
| 20 | response to type I interferon | ZBP1  STAT1 | 4.16E-05 |
|  | defense response to virus | ISG20 | 4.27E-02 |
|  | receptor signaling pathway via JAK-STAT | STAT1 | 4.75E-02 |
