# Supplementary material for: Functional genomics of AP-2α and AP-2γ in cancers: in silico study
Source: BMC Med Genomics. 2020 Nov 19;13:174. doi: 10.1186/s12920-020-00823-9 (PMC7678100; doi:10.1186/s12920-020-00823-9)
Supplement: Supplementary file 4 — Additional file 4. Detailed ontological analysis of selected modules differentiating basal-like breast cancer from other subtypes by means of AP-2α and AP-2γ lists of target genes. [file 12920_2020_823_MOESM4_ESM.docx]

**Additional file 4. Detailed ontological analysis of selected modules differentiating basal-like breast cancer from other subtypes by means of AP-2α and AP-2γ lists of target genes.**

| **Transcription factor** | **Module** | **Biological process** | **Target genes** | **p-value** |
| --- | --- | --- | --- | --- |
| **AP-2α** | **13** | protein dephosphorylation | PPP2R5E, PPP2R5C | 1.64E-02 |
|  |  | tumor necrosis factor-mediated signaling pathway | TRAF3 | 3.85E-02 |
|  |  | cellular response to stress | HSP90AA1, XRCC3, TRAF3, MNAT1, MTA1, POMT2 | 2.87E-03 |
|  | **6** | gene silencing | SRCAP, TNRC6A | 3.65E-02 |
|  |  | regulation of epidermal growth factor receptor signaling pathway | RHBDF1 | 4.71E-02 |
|  |  | regulation of autophagy | CLEC16A, SMG1 | 9.17E-03 |
|  | **12** | SMAD binding | HIPK2 | 4.63E-02 |
|  |  | actin cytoskeleton organization | ARPC1A, HIP1, LIMK1, ARPC1B | 7.50E-03 |
|  |  | intrinsic apoptotic signaling pathway by p53 class mediator | HIPK2 | 1.88E-02 |
| **AP-2γ** | **5** | Wnt signaling pathway | PCDHA6, PCDHA9, PCDHA2, PCDHA4, PCDHA11, PCDHA7, PCDHA13, PCDHA10, CSNK1E, LRP5L, PCDHA3, EP300, PCDHA8, PCDHA12, PCDHA5, PCDHA1, PCDHAC1, KREMEN1, SMARCB1 | 2.22E-12 |
|  |  | Cadherin signaling pathway | PCDHA6, PCDHA9, PCDHA2, PCDHA4, PCDHA11, PCDHA7, PCDHA13, PCDHA10, PCDHA3, PCDHA8, PCDHA12, PCDHA5, PCDHA1, PCDHAC1 | 7.00E-11 |
|  |  | Ras GTPase binding | CDC42EP1, TBC1D10A, TBC1D22A, SH3BP1 | 1.23E-02 |
|  | **13** | tumor necrosis factor-mediated signaling pathway | TRAF3 | 4.50E-02 |
|  |  | protein dephosphorylation | PPP2R5E, PPP2R5C | 2.22E-02 |
|  |  | negative regulation of apoptotic process | PSEN1, BCL2L2 | 1.68E-02 |
|  | **19** | epidermal growth factor receptor signaling pathway | PTK2B | 4.43E-02 |
|  |  | tumor necrosis factor-mediated signaling pathway | IKBKB | 2.76E-02 |
|  |  | programmed cell death | TNFRSF10B, XKR6, BNIP3L | 9.00E-03 |
